# Intranasal esketamine plus oral antidepressant for treatment-resistant depression: acute induction and maintenance relapse-prevention outcomes in a systematic review and meta-analysis

**DOI:** 10.3389/fpsyt.2026.1774549

**Published:** 2026-07-03

**Authors:** Jingqiang Xie, Chunying Pu, Mingyue Sun

**Affiliations:** Beijing University of Chinese Medicine Dongfang Hospital Qinhuangdao Hospital (Qinhuangdao Traditional Chinese Medicine Hospital), Qinhuangdao, China

**Keywords:** acute induction, esketamine, intranasal, maintenance treatment, meta-analysis, Montgomery–Åsberg Depression Rating Scale (MADRS), oral antidepressant, relapse prevention

## Abstract

Treatment-resistant depression (TRD) remains a major clinical challenge. Intranasal esketamine, used adjunctively with an oral antidepressant, has been evaluated in randomized trials, but uncertainty persists regarding the magnitude and consistency of benefit, durability, and key harms. This systematic review and meta-analysis included randomized controlled trials comparing intranasal esketamine plus an oral antidepressant versus placebo nasal spray plus the same oral antidepressant in TRD. Acute induction (≈4 weeks) and maintenance randomized-withdrawal phases were analyzed separately. Depression outcomes were assessed primarily using the Montgomery–Åsberg Depression Rating Scale (MADRS), and functional outcomes using the Sheehan Disability Scale (SDS). Two reviewers independently screened studies, extracted data, and assessed risk of bias using RoB 2.0. Random-effects models pooled mean differences (MD) for continuous outcomes, risk ratios (RR) for binary outcomes, and hazard ratios (HR) for relapse prevention. Certainty of evidence was rated using GRADE. From 1,518 records, nine reports representing six unique RCTs (1,836 participants) were included. Four acute induction RCTs (n=937) showed greater symptom reduction at day 28 with esketamine (MADRS MD −2.99, 95% CI −5.10 to −0.89; I²=48.5%). Rapid improvement was evident by day 2 (MD −3.25, 95% CI −4.65 to −1.85). Esketamine increased day-28 response (RR 1.44, 95% CI 1.20–1.74) and remission (RR 1.52, 95% CI 1.20–1.92), corresponding to approximately +154 responders and +106 remitters per 1,000 patients, respectively, based on pooled control risks. Functioning improved (SDS MD −1.70, 95% CI −2.61 to −0.79). Two maintenance randomized-withdrawal RCTs (n=899) demonstrated reduced relapse risk with continued esketamine (HR 0.51, 95% CI 0.42–0.62; I²=0%). In acute induction, esketamine increased any treatment-emergent adverse event (TEAE) (RR 1.37, 95% CI 1.25–1.50) and discontinuation due to adverse events (RR 2.68, 95% CI 1.35–5.29), with notable increases in dissociation (RR 7.33, 95% CI 4.49–11.98) and blood pressure increased events (RR 3.96, 95% CI 2.24–7.01). Maintenance TEAE rates were similar between groups (RR 1.07, 95% CI 0.99–1.17). Intranasal esketamine plus an oral antidepressant provides rapid, modest acute improvement and reduces relapse risk during maintenance among stabilized responders/remitters, but increases acute adverse events, supporting use within supervised care and individualized benefit–risk assessment.

## Introduction

Major depressive disorder (MDD) remains a leading contributor to global disability and is associated with substantial functional impairment, excess morbidity, and premature mortality (1). Although evidence-based treatments—including antidepressant pharmacotherapy, psychotherapy, and augmentation or neuromodulation strategies—benefit many individuals, approximately 20–30% of patients with MDD, and up to 30–40% of treated patients in some clinical settings depending on the operational definition, fail to achieve adequate symptom control despite multiple treatment attempts (2–7). This group is commonly described as having treatment-resistant depression (TRD), a clinical construct typically defined as nonresponse to at least two antidepressant trials of adequate dose and duration (often from different pharmacologic classes) within the current depressive episode (3–7). Recent reviews have emphasized that heterogeneity in TRD definitions, incomplete ascertainment of treatment adequacy and adherence, and variation in available treatment pathways remain major challenges for both research synthesis and clinical management (6, 7). In STAR*D, remission rates declined substantially across successive treatment steps, underscoring the diminishing returns of iterative monoaminergic approaches in routine care (2). Beyond persistent symptom burden, TRD is associated with marked reductions in quality of life, work productivity loss, and increased healthcare utilization and costs, reflecting a major unmet need at both individual and health-system levels (8).

Current guideline-endorsed strategies for inadequate antidepressant response include optimizing dose and duration, switching antidepressants, combining antidepressants, and augmentation (e.g., atypical antipsychotics, lithium, thyroid hormone), as well as structured psychotherapy and somatic treatments such as electroconvulsive therapy (ECT) and repetitive transcranial magnetic stimulation (rTMS) (9–13). However, these approaches can be limited by delayed onset of action, tolerability constraints, accessibility, and variable effectiveness in highly resistant illness. The clinical urgency is heightened by the need for treatments that act rapidly, improve functioning, and sustain benefits over time while maintaining an acceptable safety profile.

Over the past two decades, glutamatergic modulation has emerged as a promising mechanistic pathway for rapid-acting antidepressant effects. Racemic ketamine—an N-methyl-D-aspartate (NMDA) receptor antagonist—demonstrated rapid antidepressant effects in early controlled studies, catalyzing the development of related interventions aimed at TRD (14–17). Despite evidence supporting ketamine’s rapid symptom reduction, widespread implementation of intravenous ketamine is constrained by the need for supervised administration, monitoring requirements, and concerns related to dissociation, hemodynamic effects, and abuse potential. These considerations motivated investigation of esketamine, the S-enantiomer of ketamine, formulated as an intranasal therapy designed for clinic-based administration under structured safety oversight.

Intranasal esketamine has been evaluated primarily as an adjunct to a newly initiated oral antidepressant (typically an SSRI or SNRI) in short-term induction trials, followed by continuation/maintenance regimens in longer-term designs (18–25). A pivotal relapse-prevention trial used a randomized-withdrawal design in stabilized responders/remitters, assessing whether continuing esketamine plus oral antidepressant delays relapse compared with switching to placebo nasal spray while continuing the oral antidepressant (22). In parallel, older-adult populations have been studied given age-related differences in antidepressant response, medical comorbidity, and tolerability considerations (21). Importantly, intranasal esketamine’s benefit–harm profile must be interpreted in light of transient acute effects (e.g., dissociation, sedation, blood pressure increases) and trial design features such as functional unblinding and enriched maintenance populations. In the United States, esketamine is administered under a Risk Evaluation and Mitigation Strategy (REMS) with post-dose monitoring due to these safety considerations, and long-term safety has been further characterized in phase 3 open-label follow-up studies (24, 25).

Multiple evidence syntheses of esketamine have been published, generally indicating statistically significant symptom improvement versus placebo but raising questions about clinical magnitude, heterogeneity across trials and populations, and trade-offs in adverse events (26–28). Recent efforts have also leveraged individual participant data to reassess efficacy and safety signals, highlighting the importance of transparent synthesis methods and careful interpretation of absolute benefits and harms (26–28). Nevertheless, uncertainty persists regarding (i) consistency of short-term efficacy across induction trials (including older adults), (ii) durability of benefit during maintenance and relapse prevention, (iii) comparative impact on functional outcomes, and (iv) the frequency and clinical relevance of key adverse events and discontinuations.

Therefore, this systematic review and meta-analysis evaluated randomized controlled trials comparing intranasal esketamine plus oral antidepressant versus placebo nasal spray plus oral antidepressant in TRD. Consistent with the underlying clinical trial programs, acute induction (short-term symptom and functional outcomes) and randomized-withdrawal maintenance (time-to-relapse outcomes) were synthesized separately, reflecting their distinct designs and estimands. The review aimed to quantify benefits and harms across clinically relevant endpoints, explore potential sources of heterogeneity (including age-defined subgroups), and summarize the certainty of evidence using contemporary systematic review standards (29–31).

## Methods

### Protocol and reporting

This systematic review and meta-analysis was conducted and reported in accordance with the Preferred Reporting Items for Systematic Reviews and Meta-Analyses (PRISMA) 2020 statement. A review protocol was developed *a priori* to define the eligibility criteria, outcomes, and analysis plan; however, the review was not prospectively registered. The study selection process was documented using a revised PRISMA 2020 flow diagram (29).

### Eligibility criteria

Eligibility criteria were predefined using the Population–Intervention–Comparator–Outcome–Study design (PICOS) framework (32).

Population. Eligible studies enrolled adults with treatment-resistant depression (TRD), operationalized in eligible trials as inadequate response to ≥2 adequate antidepressant trials in the current episode. Trials in older adults (≥65 years) were eligible.

Intervention. The intervention of interest was intranasal esketamine administered adjunctively to a newly initiated oral antidepressant backbone at randomization. Across eligible acute trials, oral antidepressant backbones included duloxetine, escitalopram, sertraline, and venlafaxine XR, with intranasal esketamine typically dosed twice weekly during the 4-week double-blind induction period.

Comparator. Comparators were placebo nasal spray plus the same background oral antidepressant (initiated at randomization) in acute induction trials. For maintenance trials using randomized-withdrawal designs, comparators were switching to placebo nasal spray while maintaining the oral antidepressant.

Study designs. Eligible designs included:

- Acute induction double-blind parallel-group RCTs with a 4-week induction period and primary endpoint at approximately day 28.
- Maintenance (relapse prevention) randomized-withdrawal, double-blind trials conducted after open-label induction/stabilization in responders/remitters, evaluating time to relapse after randomization.

Nonrandomized studies, single-arm studies, observational designs, case series, and studies without extractable outcome data for prespecified endpoints were excluded.

### Information sources and search strategy

PubMed/MEDLINE, Embase, the Cochrane Library, PsycINFO, ClinicalTrials.gov, and the WHO ICTRP were searched from inception to December 2025. Clinical trial registries and other sources were additionally searched, reference lists of relevant reviews and included trials were screened, and conference abstracts were searched when available.

Search terms combined controlled vocabulary and keywords for “esketamine,” “intranasal,” “treatment-resistant depression,” and “randomized controlled trial,” with appropriate synonyms and Boolean operators. The complete database-specific strategies and search yields by source were prespecified and tabulated (**Supplementary Table 1**).

### Study selection

After deduplication, two reviewers independently screened titles and abstracts, followed by full-text assessment of potentially eligible reports. Disagreements were resolved by discussion and, when required, consultation with a third reviewer. Reasons for exclusion at full-text stage were recorded. The selection process was summarized in the PRISMA 2020 flow diagram.

### Data extraction and data items

Two reviewers independently extracted data using a piloted, standardized extraction form. Extracted items included:

- Trial characteristics: design (acute induction vs randomized-withdrawal maintenance), setting, blinding, follow-up duration, and sample size.
- Participant characteristics: age, sex distribution, TRD definition, and baseline depression severity (e.g., baseline MADRS).
- Intervention/comparator details: intranasal esketamine regimen (dose and frequency), oral antidepressant backbone(s), treatment duration during double-blind phases, and—when reported—backbone-specific efficacy or safety data.
- Outcomes: definitions, timepoints, measurement instruments, and safety event definitions.
- Analysis data: means/standard deviations (SDs), event counts, hazard ratios (HRs) with confidence intervals (CIs), and attrition.

When trials reported multiple publications, records were linked to the underlying unique study and the most complete dataset was extracted for each prespecified endpoint.

### Risk of bias assessment

Two reviewers independently assessed risk of bias for each included RCT using the Cochrane Risk of Bias 2 (RoB 2) tool, generating domain-level judgments (D1–D5) and an overall risk-of-bias judgment. Disagreements were resolved by consensus (33).

Because intranasal esketamine can produce acute subjective effects, heightened attention was prespecified for deviations from intended interventions, potential functional unblinding, missing outcome data patterns, and differential dropout. These considerations were explicitly reflected in RoB 2 domain judgments, consistent with the trial-level RoB synthesis presented in the results figures.

### Outcomes

Efficacy and safety outcomes were prespecified separately for acute induction and maintenance phases. Key measures included the Montgomery–Åsberg Depression Rating Scale (MADRS), Sheehan Disability Scale (SDS), Clinical Global Impression–Severity/Improvement (CGI-S/CGI-I), Clinician-Administered Dissociative States Scale (CADSS), and the Observer’s Assessment of Alertness/Sedation Scale (OAA/S) (34–38).

Primary outcomes.

1. Acute induction efficacy: change in Montgomery–Åsberg Depression Rating Scale (MADRS) score from baseline to approximately day 28 (end of double-blind induction).
2. Maintenance efficacy: time to relapse in randomized-withdrawal maintenance trials.

Key secondary efficacy outcomes (acute induction).

- Response at day 28: dichotomous outcome defined as ≥50% reduction in MADRS total score from baseline to day 28.
- Remission at day 28: dichotomous outcome defined as MADRS total score ≤10 at day 28.
- Functioning: change in Sheehan Disability Scale (SDS) total score from baseline to day 28.
- Early symptom improvement: change in MADRS score from baseline to day 2.

These endpoints and their directionality conventions (e.g., negative MD indicating greater improvement for continuous outcomes; RR >1 indicating benefit for response/remission) were prespecified and reflected in the figure definitions.

Additional efficacy outcome (acute induction).

- CGI-I responders at day 28.

### Safety outcomes

For acute induction, evaluated safety outcomes included any treatment-emergent adverse event (TEAE), discontinuation due to adverse events, any-cause discontinuation, and serious adverse events, as summarized in the acute safety forest plots.

**Figure 1:**
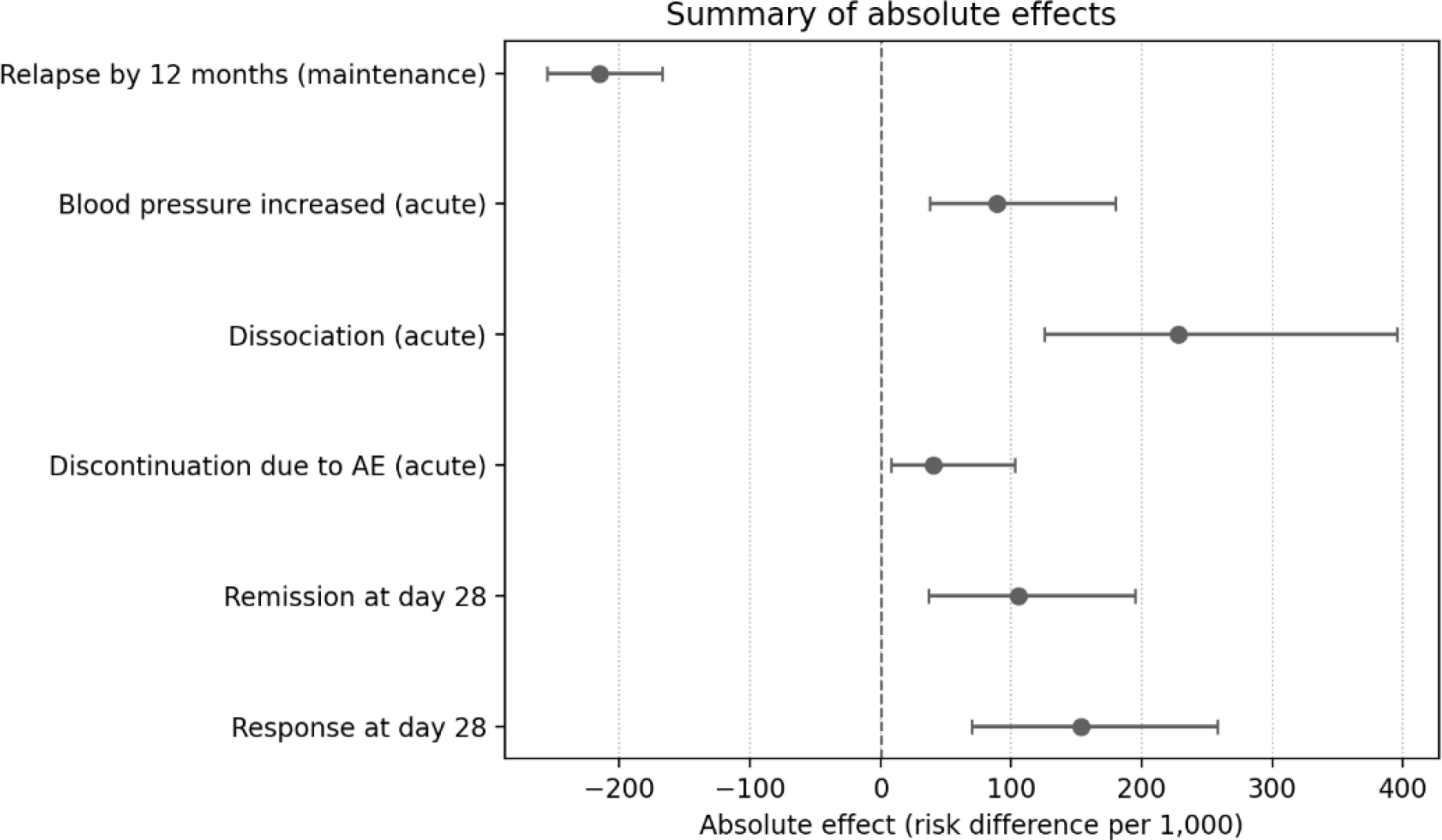
Absolute effects per 1,000 participants for key benefit–harm outcomes, derived from pooled control risks and pooled relative effects. Positive values indicate higher event rates with esketamine (beneficial for response/remission; harmful for adverse outcomes), whereas negative values indicate fewer events with esketamine (beneficial for relapse).

Common TEAEs of clinical relevance to intranasal esketamine treatment (e.g., dissociation and blood pressure increases) were also prespecified, consistent with the key safety outcomes summarized in **Table 1**, **Supplementary Table 9**, and **Figure 1**.

**Table 1:** Key safety and tolerability outcomes in acute induction and maintenance trials.

Outcome	Phase/follow-up	Participants (studies)	Esketamine group	Control group	Pooled effect (95% CI)	Interpretation
Acute induction safety (4 weeks)						
Any TEAE	Day 28	937 (4 RCTs)	78.9%	57.8%	RR 1.37 (1.25 to 1.50)	Higher with esketamine
Any-cause discontinuation	Day 28	937 (4 RCTs)	12.6%	7.7%	RR 1.62 (1.10 to 2.41)	Higher with esketamine
Discontinuation due to AE	Day 28	937 (4 RCTs)	6.4%	2.4%	RR 2.68 (1.35 to 5.29)	Higher with esketamine
Dissociation	Day 28	937 (4 RCTs)	27.2%	3.6%	RR 7.33 (4.49 to 11.98)	Higher with esketamine
Blood pressure increased	Day 28	937 (4 RCTs)	11.9%	3.0%	RR 3.96 (2.24 to 7.01)	Higher with esketamine
Maintenance safety (randomized-withdrawal)						
Any TEAE (maintenance): RR 1.07 (0.99 to 1.17); no clear difference between groups						
Any-cause discontinuation	Double-blind follow-up	899 (2 RCTs)	Lower	Higher	RR 0.53 (0.38 to 0.75)	Lower with continued esketamine

AE, adverse event; TEAE, treatment-emergent adverse event. For maintenance outcomes, pooled group percentages were not consistently reported in a directly comparable form across both trials; therefore relative effects are emphasized. For adverse outcomes, RR >1 indicates higher risk with esketamine.

For maintenance, safety outcomes mirrored the acute safety framework (any TEAE, serious adverse events, discontinuation due to adverse events, and any-cause discontinuation), assessed during double-blind randomized-withdrawal follow-up.

### Effect measures and data handling

For continuous outcomes (e.g., MADRS, SDS), change-from-baseline means and SDs were extracted where available. When change SDs were not directly reported, SDs were derived from standard errors, CIs, p-values, or other available statistics, or imputed using established methods described in the Cochrane Handbook.

Continuous outcomes were summarized using mean differences (MDs). For MADRS and SDS, negative MDs indicated greater improvement (favoring esketamine).

For binary outcomes (e.g., response, remission, TEAEs), risk ratios (RRs) were used. For benefit outcomes (response/remission/CGI-I responders), RR >1 favored esketamine. For adverse outcomes, RR >1 indicated higher risk with esketamine.

For time-to-event outcomes (maintenance relapse prevention), hazard ratios (HRs) and their precision estimates were extracted, prioritizing intention-to-treat analyses and Cox model estimates where available. When HRs were not explicitly reported, derivation of log(HR) and standard errors using established summary-data methods was planned.

### Data synthesis and statistical analysis

Meta-analyses were conducted when ≥2 studies reported sufficiently comparable data for a given outcome and timepoint.

Primary synthesis model. Random-effects meta-analyses were performed to account for between-study heterogeneity. Effect estimates were pooled using inverse-variance weighting. For the between-study variance (τ²), the DerSimonian–Laird (DL) estimator was used for the primary random-effects analyses. As sensitivity analyses, the primary meta-analyses were repeated using alternative τ² estimators (e.g., REML), Hartung–Knapp adjustments, and fixed-effect models. Heterogeneity was summarized with I², τ², and Cochran’s Q, and 95% prediction intervals were reported where appropriate (39–41).

Statistical significance. Two-sided tests with α=0.05 were used.

Software. Primary analyses were conducted using R (packages such as metafor and meta), with Bayesian sensitivity analyses conducted using Bayesian random-effects routines (e.g., bayesmeta) and trial sequential analyses performed using dedicated TSA software (42–44).

### Subgroup analyses

Clinically motivated subgroup analyses were prespecified to explore effect modification:

- Age subgroup (acute induction): trials enrolling adults 18–64 years versus trials enrolling older adults ≥65 years, given potentially different treatment response and tolerability.
- Maintenance stabilization status: stable remission versus stable response at randomization in randomized-withdrawal trials, consistent with maintenance trial populations and relapse risk stratification.

Subgroup differences were assessed using interaction tests (between-subgroup heterogeneity).

### Sensitivity analyses and robustness checks

To evaluate robustness of pooled estimates, prespecified sensitivity analyses included fixed-effect models, Hartung–Knapp adjustment for random-effects inference, alternative τ² estimators, leave-one-out analyses, and Bayesian random-effects meta-analysis (45–49).

### Small-study effects and publication bias

Small-study effects and potential publication bias were assessed using funnel plots when feasible. Exploratory Egger’s regression test and Begg’s rank correlation test were additionally performed, and the trim-and-fill method was applied as an exploratory adjustment approach, recognizing limited power when few trials contributed to an outcome. For outcomes supported by very few studies (e.g., maintenance relapse prevention with two randomized-withdrawal trials), formal publication-bias testing was not performed due to lack of interpretability (50–52).

### Trial sequential analysis

To evaluate the risk of random errors and the sufficiency of cumulative evidence, trial sequential analysis (TSA) was performed for the primary acute outcome (MADRS change at day 28). Parameters included a two-sided α=0.05 and power (1−β)=80%, with required information size derived based on anticipated effect size and observed heterogeneity. TSA findings were incorporated as part of the robustness assessment (53, 54).

### Certainty of evidence

Certainty of evidence for prespecified key outcomes was rated using the GRADE approach. To enhance interpretability, pooled relative effects were translated into absolute effects per 1,000 participants using assumed baseline risks in the control group and displayed in an absolute-effects plot aligned with the key efficacy and safety outcomes (30, 31).

### Ethics and patient/public involvement

Ethics approval was not required because this study synthesized data from published reports and trial registries without individual participant data. No patients or members of the public were involved in the design, conduct, or reporting of this review.

## Results

For clarity, the main characteristics of included trials are summarized in **Table 2**, pooled efficacy outcomes in **Table 3**, and key safety/tolerability outcomes in **Table 1**.

**Table 2:** Key characteristics of included randomized controlled trials (intranasal esketamine plus oral antidepressant for treatment−resistant depression).

Study (source)	Design/phase	Population (TRD definition)	Sample size (E/C)	Mean age, years	Female, %	Baseline MADRS*	Oral AD backbone (initiated at randomization)	Esketamine regimen	Double-blind follow-up	Primary endpoint
Trial A (2019) (19)	Multicenter, double-blind, parallel-group; acute induction	Adults 18–64; inadequate response to ≥2 adequate AD trials in current episode	172/174	45.9	63	38.1	Duloxetine/escitalopram/sertraline/venlafaxine XR	56 mg first dose; flexible 56/84 mg twice weekly × 4 weeks	4 weeks	MADRS change day 28
Trial B (2019) (20)	Double-blind, parallel-group; acute induction	Adults 18–64; ≥2 failed AD trials (current episode)	114/109	44.8	60	37.4	Duloxetine/escitalopram/sertraline/venlafaxine XR	Fixed-dose 84 mg twice weekly × 4 weeks†	4 weeks	MADRS change day 28
Trial C (2023) (23)	Double-blind, parallel-group; acute induction	Adults 18–64; ≥2 failed AD trials (current episode)	115/115	45.5	62	37.8	Duloxetine/escitalopram/sertraline/venlafaxine XR	Flexible 56/84 mg twice weekly × 4 weeks	4 weeks	MADRS change day 28
Trial D (2020) (21)	Double-blind, parallel-group; acute induction	Older adults ≥65; ≥2 failed AD trials (current episode)	69/69	68.3	58	36.9	Duloxetine/escitalopram/sertraline/venlafaxine XR	Start 28 mg; flexible 28/56/84 mg twice weekly × 4 weeks	4 weeks	MADRS change day 28
Trial E (2020) (25)	RW, double-blind maintenance after open-label induction/stabilization	Responders/remitters after ~16 weeks esketamine + oral AD	301/302	46.0	64	9.8‡	Same oral AD continued	Continue esketamine weekly → q2w (protocol-guided) vs placebo spray	Up to 48 weeks	Time to relapse
Trial F (2019) (22)	RW, double-blind maintenance	Stable responders/remitters after induction/stabilization	149/147	47.1	60	10.6‡	Same oral AD continued	Continue esketamine q1–2w vs placebo spray	Up to 32 weeks	Time to relapse

AD, antidepressant; MADRS, Montgomery–Åsberg Depression Rating Scale; RW, randomized withdrawal; E, esketamine nasal spray + oral AD; C, placebo nasal spray + oral AD.

*Baseline MADRS refers to randomization baseline for acute parallel−group trials; for RW trials, baseline MADRS refers to maintenance randomization (post−stabilization).

‡Lower scores reflect entry into randomized maintenance among stable responders/remitters. Duration of illness was not reported consistently across the included trial publications and therefore could not be tabulated uniformly.

**Table 3:** Pooled primary and secondary efficacy outcomes in acute induction and maintenance trials.

Outcome	Scale/endpoint	Follow-up	Participants (studies)	Pooled effect (95% CI)	I² (%)	Interpretation
Acute induction						
Depressive symptoms (primary outcome)	MADRS change from baseline	Day 28	937 (4 RCTs)	MD −2.99 (−5.10 to −0.89)	48.5	Negative MD favors esketamine
Early symptom improvement	MADRS change from baseline	Day 2	937 (4 RCTs)	MD −3.25 (−4.65 to −1.85)	3.0	Negative MD favors esketamine
Clinical response	≥50% MADRS reduction	Day 28	937 (4 RCTs)	RR 1.44 (1.20 to 1.74)	27.1	RR >1 favors esketamine
Remission	MADRS ≤10	Day 28	937 (4 RCTs)	RR 1.52 (1.20 to 1.92)	0	RR >1 favors esketamine
Functional improvement	SDS total change	Day 28	937 (4 RCTs)	MD −1.70 (−2.61 to −0.79)	0	Negative MD favors esketamine
Global improvement	CGI-I responders	Day 28	937 (4 RCTs)	RR 1.33 (1.16 to 1.53)	0.4	RR >1 favors esketamine
Maintenance randomized-withdrawal						
Relapse prevention	Time to relapse	6–12 months	899 (2 RCTs)	HR 0.51 (0.42 to 0.62)	0	HR <1 favors continued esketamine

CGI-I, Clinical Global Impression–Improvement; HR, hazard ratio; MADRS, Montgomery–Åsberg Depression Rating Scale; MD, mean difference; RR, risk ratio; SDS, Sheehan Disability Scale. For continuous outcomes, negative MD values favor esketamine; for response/remission/CGI-I, RR >1 favors esketamine; for relapse prevention, HR <1 favors continued esketamine.

### Study selection

**Figure 2:**
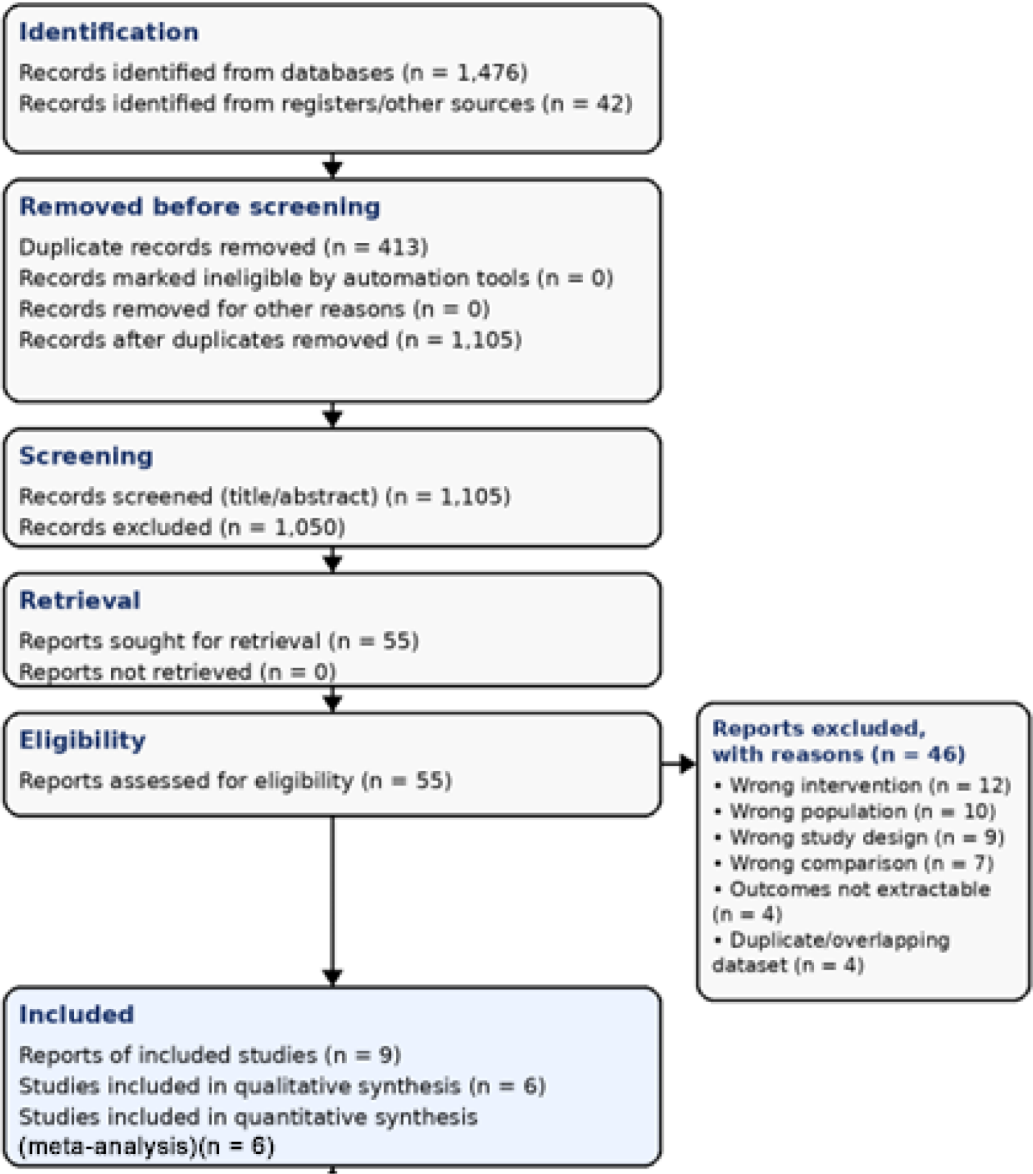
Revised PRISMA 2020 flow diagram of study identification and selection. Records denote database entries/citations, reports denote full-text articles, and studies denote unique trials. Full-text exclusion reasons are shown in the right-hand panel.

We identified 1,518 records (1,476 from databases and 42 from trial registries/other sources) (**Figure 2**; **Supplementary Table 1**). After removal of 413 duplicates, 1,105 records were screened and 1,050 were excluded at the title/abstract stage. Fifty-five reports were sought for retrieval and all were retrieved for full-text assessment; 46 reports were excluded with prespecified reasons, leaving nine reports representing six unique randomized controlled trials (1,836 participants) for inclusion in the review and meta-analysis (**Figure 2**; **Table 2**; **Supplementary Tables 1**, **2**).

### Study characteristics

Six randomized controlled trials were included: four short-term double-blind, parallel-group acute induction RCTs and two double-blind randomized-withdrawal maintenance RCTs (**Table 2**; **Supplementary Figure 1**). The acute induction trials enrolled 937 participants and evaluated intranasal esketamine administered twice weekly for 4 weeks in combination with a newly initiated oral antidepressant; three trials recruited adults aged 18–64 years and one trial focused on older adults aged ≥65 years (**Table 2**; **Supplementary Figure 1**). The maintenance trials enrolled 899 participants at the maintenance randomization baseline and used randomized-withdrawal designs after open-label induction/stabilization, randomizing stable responders/remitters to continue esketamine or discontinue to placebo nasal spray while maintaining the oral antidepressant backbone (**Table 2**; **Supplementary Figure 1**).

Across acute induction RCTs, baseline depression severity was high (weighted mean baseline MADRS 37.7), the weighted mean age was 49.0 years, and 61.3% of participants were female (**Supplementary Table 5**). Participants initiated a protocol-specified oral antidepressant at randomization (duloxetine, escitalopram, sertraline, or venlafaxine XR), with duloxetine being the most frequently used backbone (**Supplementary Table 3**). Esketamine dosing was flexible (56/84 mg) in three acute trials, one trial used a predominantly fixed 84 mg schedule, and the older-adult trial typically started at 28 mg with protocol-guided titration (**Table 2**; **Supplementary Table 4**).

In maintenance RCTs, symptom severity at maintenance randomization was low (weighted mean MADRS 10.1), reflecting the enriched population of stabilized responders/remitters (**Supplementary Table 5**). Primary endpoints were MADRS change at day 28 for acute induction trials and time to relapse for maintenance trials (**Table 2**).

### Background oral antidepressants

The protocol-specified oral antidepressant backbones initiated at randomization were duloxetine (289/937, 30.8%), escitalopram (235/937, 25.1%), sertraline (222/937, 23.7%), and venlafaxine XR (191/937, 20.4%) (**Supplementary Table 3**). Published RCT reports did not provide efficacy or safety outcomes stratified by the individual oral antidepressant backbone in sufficient detail to support a valid head-to-head comparison.

### Risk of bias

**Figure 3:**
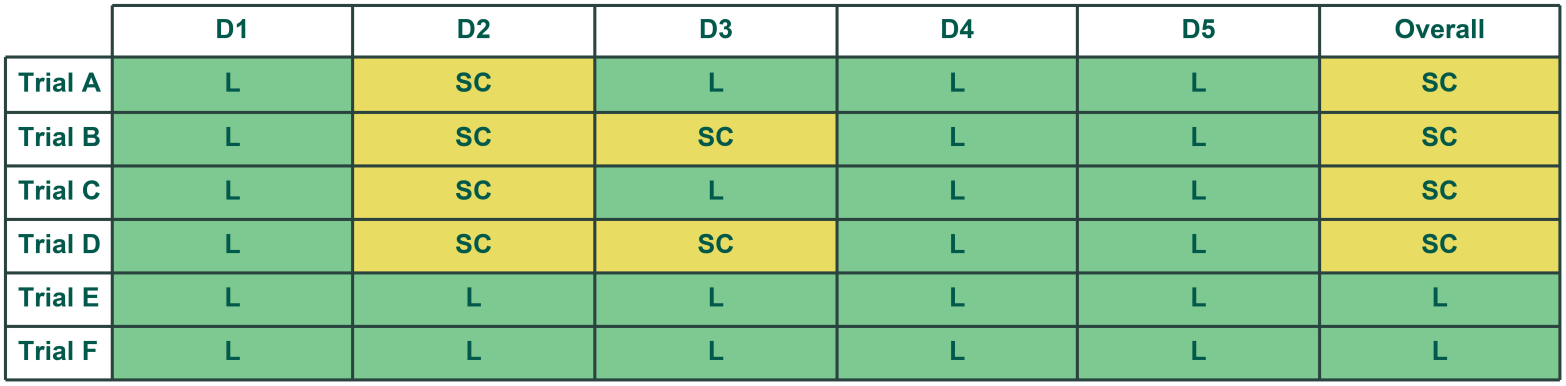
Risk of bias assessment of included randomized controlled trials using the Cochrane RoB 2.0 tool. Domain-level judgements (D1–D5) and the overall risk-of-bias judgement are shown for each trial (Trials A–F). “Some concerns” in acute trials primarily reflect the potential for functional unblinding and deviations from intended interventions due to distinctive acute subjective effects, and (in selected trials) differential missing outcome data. D1, randomization process; D2, deviations from intended interventions; D3, missing outcome data; D4, measurement of the outcome; D5, selection of the reported result.

Risk of bias was assessed at the outcome level using the Cochrane RoB 2.0 tool (**Figure 3**; **Supplementary Table 6**). For acute induction trials, judgements were anchored to the primary efficacy outcome (MADRS change at day 28); for maintenance randomized−withdrawal trials, judgements were anchored to time to relapse (**Supplementary Table 6**). Overall, 2/6 trials (33.3%) were judged at low risk of bias and 4/6 (66.7%) had some concerns; no trial was rated high risk (**Figure 3**; **Supplementary Figure 2**; **Supplementary Table 6**). In acute trials, the most common concern related to deviations from intended interventions (RoB2 Domain 2), reflecting potential functional unblinding due to distinctive acute subjective effects (e.g., dissociation/sedation) (**Figure 3**; **Supplementary Table 6**). A second contributor was missing outcome data (Domain 3) in two acute trials, where discontinuations and missing primary endpoint values were numerically higher in the esketamine group (**Supplementary Table 7**; **Supplementary Table 6**). Both maintenance trials were judged low risk across RoB2 domains (**Figure 3**; **Supplementary Table 6**).

### Efficacy (acute induction)

**Figure 4:**
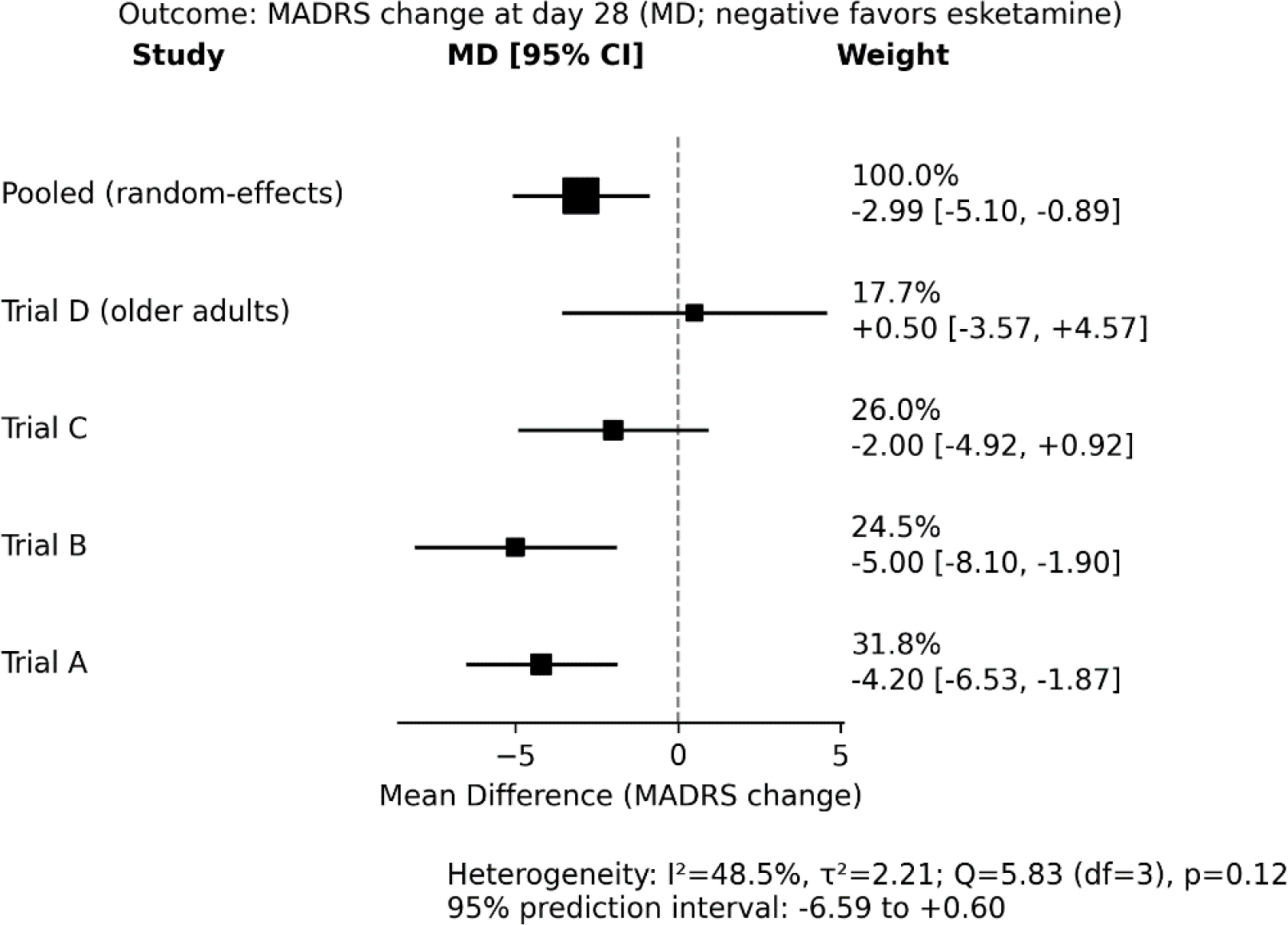
Acute induction: MADRS change from baseline to day 28 (random-effects meta-analysis). Random-effects meta-analysis of mean difference (MD) in MADRS change from baseline to day 28 comparing intranasal esketamine plus oral antidepressant versus placebo nasal spray plus oral antidepressant in acute induction randomized controlled trials. Negative MD values indicate greater symptom improvement with esketamine. Study weights are from the random-effects model. Heterogeneity statistics (I², τ², Q) and the 95% prediction interval are shown.

Four double-blind acute induction RCTs (n=937) contributed to the acute-phase efficacy meta-analyses (**Table 2**). Intranasal esketamine plus a newly initiated oral antidepressant was associated with a greater reduction in depressive symptoms at day 28 than placebo nasal spray plus the same oral antidepressant (random-effects MD in MADRS change: −2.99 points, 95% CI −5.10 to −0.89; I²=48.5%) (**Figure 4**; **Table 3**). The 95% prediction interval ranged from −6.59 to +0.60, indicating that the magnitude of benefit may vary across settings and populations (**Figure 4**). Heterogeneity appeared to be largely driven by the single older-adult trial (≥65 years), which showed an effect estimate close to null (**Figure 4**; **Supplementary Table 10**; **Supplementary Figure 5**).

**Figure 5:**
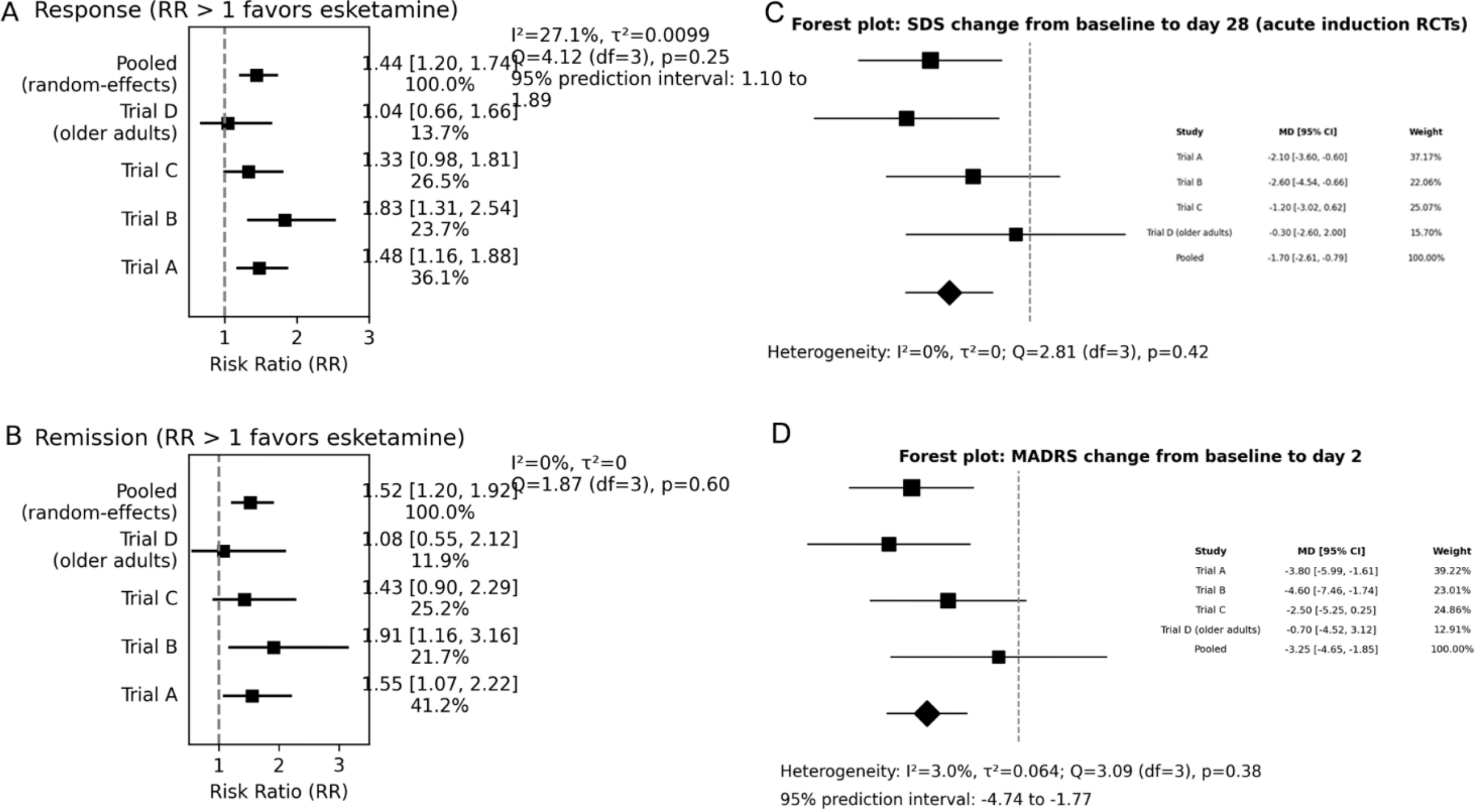
Acute induction efficacy outcomes: **(A)** response at day 28, **(B)** remission at day 28, **(C)** SDS change at day 28, and **(D)** MADRS change at day 2. Random-effects meta-analyses of day-28 clinical response **(A)** and remission **(B)** comparing intranasal esketamine plus oral antidepressant versus placebo nasal spray plus oral antidepressant. Effect sizes are expressed as risk ratios (RR) with 95% confidence intervals; RR values >1 favor esketamine. Domain-specific heterogeneity statistics are shown beneath each panel, and a 95% prediction interval is provided where heterogeneity is present. Random-effects meta-analysis of mean difference (MD) in Sheehan Disability Scale (SDS) total score change from baseline to day 28 comparing intranasal esketamine plus oral antidepressant versus placebo nasal spray plus oral antidepressant in acute induction randomized controlled trials. Negative MD indicates greater improvement in functional impairment with esketamine. Study weights reflect the random-effects model **(C)**. Random-effects meta-analysis of mean difference (MD) in MADRS change from baseline to day 2 comparing intranasal esketamine plus oral antidepressant versus placebo nasal spray plus oral antidepressant. Negative MD indicates greater early symptom improvement with esketamine **(D)**.

At day 28, esketamine increased the likelihood of response and remission compared with control (response: RR 1.44, 95% CI 1.20–1.74, I²=27.1%; remission: RR 1.52, 95% CI 1.20–1.92, I²=0%) (**Figures 5A, B**; **Table 3**). Using pooled control risks, this corresponds to an absolute increase of +154 responders per 1,000 (95% CI+70 to +258) and +106 remitters per 1,000 (95% CI+41 to +187) (**Figure 1**).

**Figure 6:**
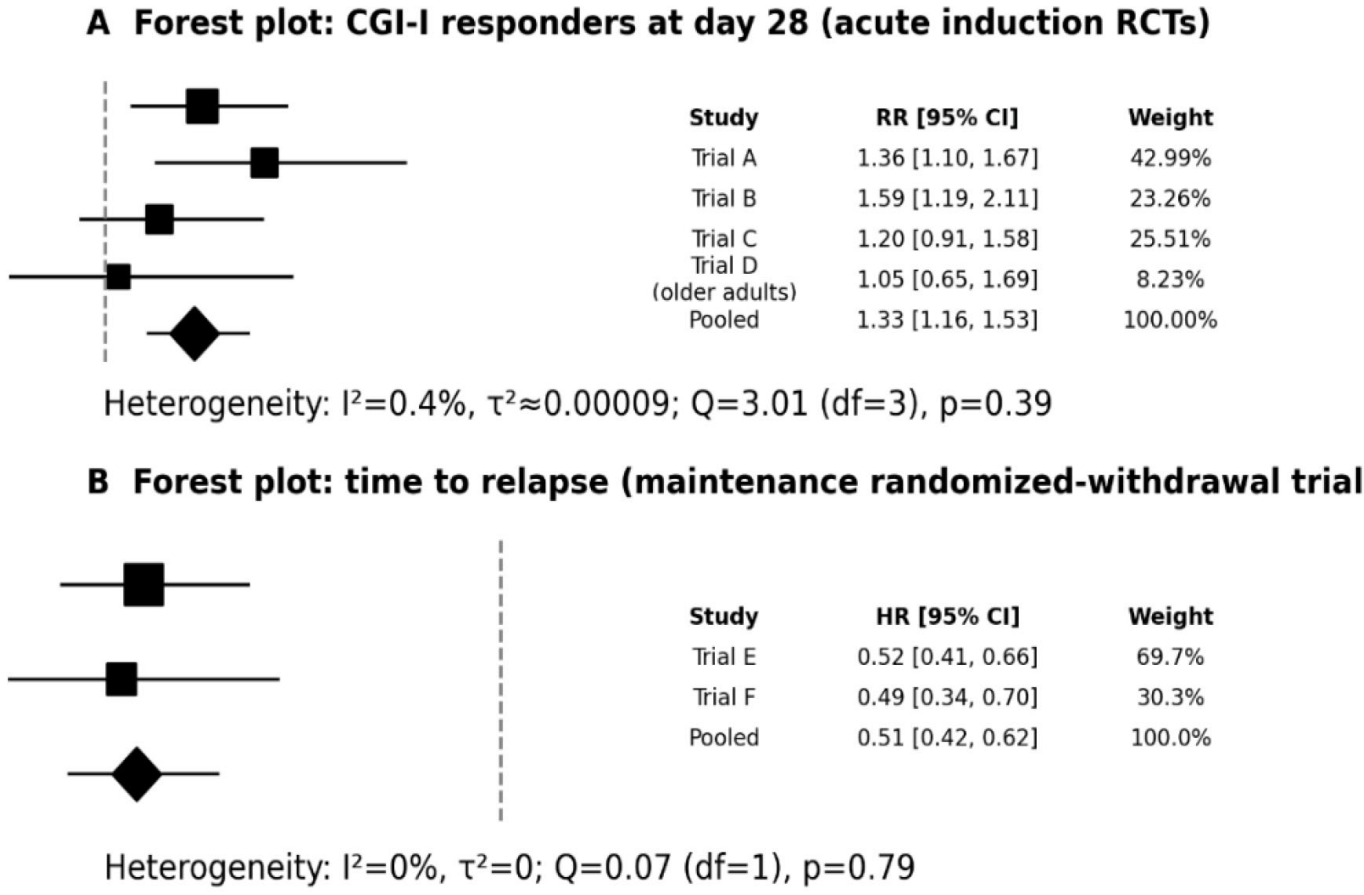
**(A)** CGI-I responders at day 28 (acute induction) and **(B)** time to relapse in randomized-withdrawal maintenance trials. Random-effects meta-analysis of CGI-I responder rates at day 28 comparing intranasal esketamine plus oral antidepressant versus placebo nasal spray plus oral antidepressant. Effect sizes are expressed as risk ratios (RR) with 95% confidence intervals; RR >1 indicates higher likelihood of clinician-rated global improvement with esketamine **(A)**. Random-effects meta-analysis of hazard ratios (HR) for time to relapse in randomized-withdrawal maintenance trials. HR <1 indicates reduced relapse risk with continued intranasal esketamine plus oral antidepressant compared with switching to placebo nasal spray plus oral antidepressant. Study weights are from the random-effects model; heterogeneity statistics are shown **(B)**.

Functional outcomes favored esketamine, with greater improvement in SDS total score at day 28 (MD −1.70, 95% CI −2.61 to −0.79; I²=0%) (**Figure 5C**; **Table 3**). Rapid antidepressant effects were also observed by day 2 (MADRS change: MD −3.25, 95% CI −4.65 to −1.85; I²=3.0%; 95% prediction interval −4.74 to −1.77) (**Figure 5D**; **Table 3**). Clinician-rated global improvement (CGI-I responders at day 28) was more frequent with esketamine (RR 1.33, 95% CI 1.16–1.53; I²=0.4%) (**Figure 6A**; **Table 3**).

In prespecified subgroup analyses, adult trials consistently favored esketamine, whereas the older-adult trial did not demonstrate benefit; the interaction test for subgroup differences was borderline for MADRS change (p=0.055) (**Supplementary Table 10**; **Supplementary Figures 5**, **6**).

### Maintenance/relapse prevention

Two randomized-withdrawal (RW) maintenance trials (Trials E and F; total n=899) evaluated relapse prevention in participants who achieved response/remission after an open-label induction/stabilization phase of intranasal esketamine plus an oral antidepressant, and were then randomized to continue esketamine or switch to placebo nasal spray while continuing the same oral antidepressant (**Table 2**; **Supplementary Figure 1**). At maintenance randomization, symptom severity was low (baseline MADRS 9.8 in Trial E and 10.6 in Trial F), and double-blind follow-up lasted up to 48 weeks (Trial E) and 32 weeks (Trial F) (**Table 2**; **Supplementary Table 5**; **Figure 6B**).

Continuing esketamine plus oral antidepressant significantly reduced the hazard of relapse compared with switching to placebo nasal spray plus oral antidepressant (random-effects: HR 0.51, 95% CI 0.42–0.62; p<0.001; I²=0%, τ²=0) (**Figure 6B**; **Table 3**). Trial-level relapse proportions were 25.9% (78/301) versus 44.0% (133/302) in Trial E and 22.1% (33/149) versus 39.5% (58/147) in Trial F; corresponding trial HRs were 0.52 and 0.50, respectively (**Figure 6B**).

Using an assumed control relapse risk of 440 per 1,000 over 6–12 months, the estimated relapse risk with continued esketamine was 224 per 1,000 (absolute risk reduction 216 per 1,000; moderate certainty) (**Figure 1**; **Supplementary Table 9**). In prespecified subgroup analyses, relapse prevention was observed both among participants in stable remission at maintenance randomization (pooled HR 0.45, 95% CI 0.36–0.56) and among stable responders (non-remitters) (pooled HR 0.57, 95% CI 0.46–0.70), with no evidence of subgroup interaction (p=0.30) (**Supplementary Table 11**; **Supplementary Figure 7**).

### Safety and tolerability

**Figure 7:**
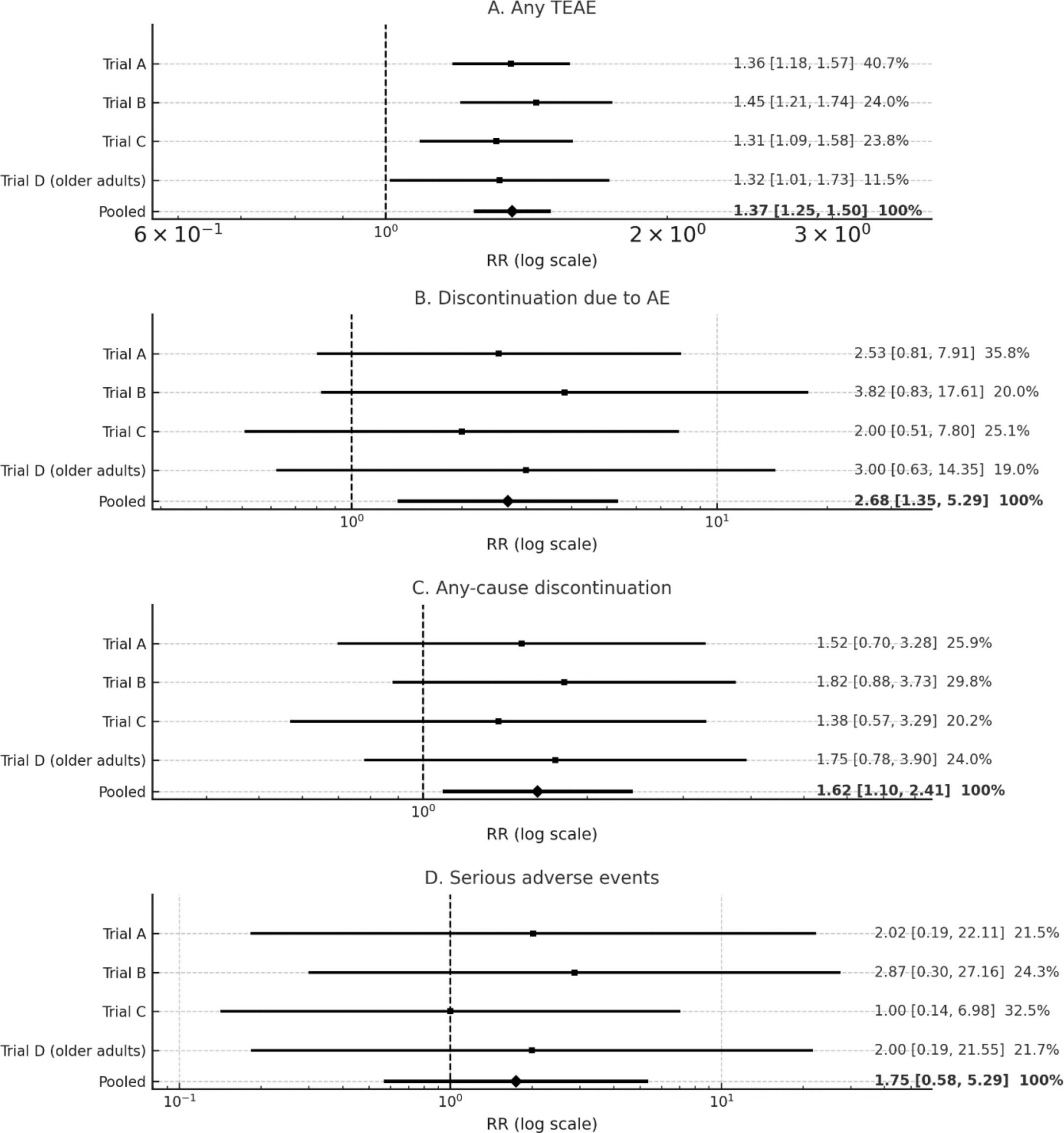
Acute induction safety outcomes: any TEAE, discontinuation due to AE, any-cause discontinuation, and serious adverse events (random-effects meta-analyses). Overall safety and tolerability outcomes (acute induction). Panels show random-effects meta-analyses (risk ratios, RR) comparing intranasal esketamine plus oral antidepressant versus placebo nasal spray plus oral antidepressant for: **(A)** any treatment-emergent adverse event (TEAE), **(B)** discontinuation due to adverse events, **(C)** any-cause discontinuation, and **(D)** serious adverse events. Squares represent study-specific RRs with size proportional to inverse-variance weight; horizontal lines indicate 95% CIs; diamonds indicate pooled effects. The vertical dashed line marks RR = 1.0 (no difference).

Acute induction (4 weeks). Across four double-blind induction trials (esketamine plus oral antidepressant, n=470; placebo nasal spray plus oral antidepressant, n=467), treatment-emergent adverse events (TEAEs) were more frequent with esketamine (**Figure 7**; **Table 1**). Any TEAE occurred in 78.9% versus 57.8% (RR 1.37, 95% CI 1.25–1.50). Discontinuation due to adverse events was higher with esketamine (6.4% versus 2.4%; RR 2.68, 95% CI 1.35–5.29), as was any-cause discontinuation (12.6% versus 7.7%; RR 1.62, 95% CI 1.10–2.41). Serious adverse events were uncommon in both groups (1.9% versus 1.1%; RR 1.75, 95% CI 0.58–5.29).

Neurological/perceptual symptoms and blood-pressure-related events were more frequent with esketamine, including dissociation (27.2% versus 3.6%; RR 7.33, 95% CI 4.49–11.98), dizziness (RR 2.60, 95% CI 1.83–3.69), somnolence (RR 2.89, 95% CI 1.88–4.43), nausea (18.7% versus 10.3%), and investigator-reported “blood pressure increased” events (11.9% versus 3.0%; RR 3.96, 95% CI 2.24–7.01) (**Supplementary Figure 3**; **Table 1**).

Key harms are summarized in **Table 1** and **Figure 1**, including discontinuation due to adverse events (+40/1,000), dissociation (+228/1,000), and blood pressure increased events (+89/1,000).

Exploratory age-stratified analyses did not show clear effect modification for safety outcomes (interaction p-values 0.27–0.87) (**Supplementary Table 8**).

Maintenance/relapse prevention. In two randomized-withdrawal maintenance trials (continued esketamine plus antidepressant, n=450; placebo nasal spray plus antidepressant, n=449), overall TEAE rates were high in both groups and not clearly different (RR 1.07, 95% CI 0.99–1.17) (**Supplementary Figure 4**; **Table 1**). Serious adverse events (RR 1.00, 95% CI 0.47–2.13) and discontinuation due to adverse events (RR 1.30, 95% CI 0.74–2.29) were also similar, whereas any-cause discontinuation was lower with continued esketamine (RR 0.53, 95% CI 0.38–0.75) (**Supplementary Figure 4**; **Table 1**).

### Robustness and small-study effects

Sensitivity analyses supported the robustness of the acute induction findings; detailed results are provided in **Supplementary Tables 12**, **13** and **Supplementary Figure 8**. For the primary outcome (MADRS change from baseline to day 28), the pooled benefit remained consistently in favor of intranasal esketamine plus an oral antidepressant across alternative analytic choices (fixed-effect vs random-effects, alternative τ² estimator, Hartung–Knapp adjustment, endpoint vs change-score analysis, standardized effect size, and conservative SD assumptions) (**Supplementary Table 12**). Excluding the older-adult trial reduced heterogeneity and yielded a slightly larger effect estimate (**Supplementary Table 12**; **Supplementary Figure 5**). A leave-one-out influence analysis indicated that no single acute trial unduly drove the pooled MADRS estimate (**Supplementary Figure 8**). Response and remission effects at day 28 were similarly stable in leave-one-out and other sensitivity analyses (**Supplementary Table 13**). An exploratory Bayesian random-effects meta-analysis produced a comparable estimate for MADRS change (posterior mean MD −3.02; 95% credible interval −5.44 to −0.63), with a high posterior probability of benefit (**Supplementary Table 14**).

In exploratory trial sequential analysis (TSA), the required information size was approximately 1200 participants; the cumulative Z-curve crossed both the conventional and TSA monitoring boundaries before reaching the required information size, suggesting that—under the prespecified assumptions—the acute symptom benefit may be robust, although additional trials could further refine the precision of the estimate (**Supplementary Figure 9**). Visual inspection of funnel plots for MADRS change and response at day 28 did not suggest marked asymmetry; however, given the small number of acute trials, statistical tests were treated as exploratory (**Supplementary Figures 10**, **11**). Consistent with this, exploratory Egger/Begg testing did not indicate statistically significant small-study effects and trim-and-fill did not impute missing studies across major acute outcomes (**Supplementary Table 15**). For maintenance relapse-prevention outcomes, only two randomized-withdrawal trials contributed, precluding meaningful assessment of small-study effects or publication bias (**Table 2**; **Figure 6B**).

### Certainty of evidence (GRADE)

Certainty of evidence was assessed using GRADE for prespecified key outcomes and is summarized in **Supplementary Table 9** and **Supplementary Figure 12**. Overall certainty was moderate for the primary continuous outcome (MADRS change at day 28), remission, functioning (SDS change at day 28), relapse prevention, and discontinuation due to adverse events, and high for response at day 28, early symptom improvement (MADRS change at day 2), dissociation, and blood pressure increased events.

Absolute effects per 1,000 patients, derived from pooled control risks and pooled relative effects, are visualized in **Figure 1**. In acute induction, esketamine plus an oral antidepressant improved depressive symptoms and functioning, increased response and remission, and increased several clinically relevant adverse events; in maintenance trials, continued esketamine reduced relapse risk with no clear increase in overall TEAEs (**Tables 1**, **3**; **Figure 1**; **Supplementary Table 9**).

## Discussion

This review indicates that adjunctive intranasal esketamine provides rapid but modest acute symptom improvement, improves functional outcomes, and supports relapse prevention among stabilized responders/remitters. These benefits should be weighed against the higher acute burden of adverse events, particularly dissociation and blood-pressure increases.

### Interpretation of the main findings

In adults with TRD, the acute efficacy signal is clinically relevant because eligible patients had already failed multiple adequate antidepressant trials; however, the average effect remains modest and should not be interpreted as a uniformly large response in all treated patients. The early separation from control by day 2 is consistent with the rapid-acting glutamatergic mechanism proposed for ketamine-derived agents and may be relevant when prompt symptom reduction is clinically important.

Heterogeneity and population signal. The observed heterogeneity in day-28 MADRS outcomes was largely driven by the older-adult trial, and the prediction interval included the null (−6.59 to +0.60), suggesting that the average benefit may not be uniform across settings and patient subgroups. This interpretation aligns with TRANSFORM-3, which did not meet statistical significance for the primary endpoint overall and showed larger effects in younger elderly subgroups (65–74) than in patients ≥75 years (21). Clinically, these findings support a more nuanced view: while esketamine augmentation can provide meaningful benefit for some patients with TRD, patient selection (including age, frailty, and comorbidity burden) likely influences the expected magnitude of effect.

Maintenance efficacy. The maintenance findings are best understood as evidence for continuing esketamine in patients who have already responded or remitted and tolerated induction, rather than as proof of long-term benefit in all patients starting esketamine. This distinction is important because randomized-withdrawal designs answer a relapse-prevention question in an enriched population, not an initiation question in an unselected TRD population (22).

Background oral antidepressants. The concomitant oral antidepressants in the acute RCT program were limited to protocol-specified SSRIs/SNRIs (duloxetine, escitalopram, sertraline, and venlafaxine XR). Because backbone-specific efficacy and safety outcomes were not reported in a way that allowed a valid comparative meta-analysis, the present review supports the adjunctive effect of intranasal esketamine combined with a newly initiated oral antidepressant strategy at the class/program level, but it does not establish superiority of any individual oral antidepressant backbone.

### Benefit–harm balance and implications for practice

A central question for clinicians is whether the symptomatic and relapse-prevention benefits justify the safety and tolerability burden.

Absolute benefits. Translation into absolute effects suggests that the incremental probability of response and remission may be clinically meaningful in a TRD population, especially because symptom improvement can emerge early. These absolute estimates should be interpreted alongside adverse-event risks, monitoring burden, cost, access, and patient preferences.

Short-term adverse effects. The acute safety profile is dominated by transient neurological/perceptual symptoms and hemodynamic events. Although many events are short-lived, they justify supervised dosing, blood-pressure monitoring, post-dose observation, and careful counselling about dissociation, sedation, dizziness, nausea, and driving restrictions, consistent with prescribing-information safety precautions (24).

Maintenance safety. Among patients who tolerate induction and enter maintenance, continuation appears feasible, with no clear excess in overall TEAEs in the randomized-withdrawal evidence. However, maintenance evidence remains limited, and longer real-world follow-up is needed to characterize sustained safety, adherence, cognition, functioning, and healthcare-resource implications beyond controlled trial environments.

### Relation to the existing evidence base

Our findings are concordant with the direction and general magnitude of effect reported in pivotal trials of adjunctive intranasal esketamine in TRD and the relapse-prevention study (18–23). The synthesis extends individual trial reports by separating acute induction from maintenance estimands and by presenting absolute benefit–harm estimates alongside GRADE certainty assessments.

Recent secondary evidence has likewise concluded that intranasal esketamine improves depressive symptoms and response outcomes in TRD while increasing acute adverse effects such as dizziness and nausea, which is consistent with our safety synthesis (26–28). Our findings are also directionally consistent with the multicenter REAL-ESK study, which reported effectiveness and acceptable tolerability in routine clinical practice, although direct comparison is limited by its observational design and more heterogeneous patient population (55). Taken together, the available evidence suggests that adjunctive esketamine provides incremental benefit over switching to a new oral antidepressant alone, but with a benefit–risk profile that is best optimized when care pathways include structured monitoring and counseling on transient psychoactive effects.

### Strengths of this review

Several design features strengthen the interpretability of our synthesis. First, we stratified evidence by acute induction versus maintenance relapse prevention, reflecting how esketamine is used in practice (initiation followed by continuation decisions). Second, we combined conventional pairwise meta-analysis with robustness checks (leave-one-out, Bayesian sensitivity, and trial sequential analysis), which consistently supported the direction of benefit while highlighting remaining imprecision for some outcomes. Third, our GRADE assessments were largely moderate to high certainty for key acute efficacy and relapse outcomes, aiding clinical translation. The review was developed in line with contemporary systematic review reporting standards (PRISMA 2020) and evidence-certainty frameworks (GRADE) (29–31).

### Limitations and remaining uncertainties

This review also has important limitations that affect generalizability and certainty for some endpoints.

Blinding and risk of bias. Although most trials were judged low risk or with some concerns, several had “some concerns” primarily due to potential functional unblinding (given acute psychoactive effects) and missing outcome data. Such issues may bias subjective symptom outcomes in either direction.

Limited number of trials and participants for some outcomes. While the total sample was substantial, several secondary outcomes were informed by only 2–3 trials, limiting precision and the ability to formally explore publication bias or moderators (e.g., baseline severity, comorbidity, antidepressant class, or dosing strategy).

Background antidepressant comparison. Although all acute RCTs used a newly initiated oral antidepressant backbone, the published reports did not provide efficacy or safety outcomes stratified by duloxetine, escitalopram, sertraline, or venlafaxine XR. Consequently, our review cannot determine whether the benefit–harm profile differed by background antidepressant.

Short induction duration and limited long-term comparative data. Induction trials largely ended at 4 weeks, while maintenance trials relied on designs that include enrichment (randomized withdrawal of responders/remitters) (22). Enriched designs are appropriate for relapse prevention questions but may overestimate effectiveness if applied to an unselected TRD population initiating treatment.

Population gaps. The signal of smaller or uncertain benefit in older adults (and potential age-related heterogeneity) underscores the need for more targeted evidence for late-life TRD, including patients ≥75 years and those with higher medical comorbidity burdens (21).

Protocol registration. The review protocol was developed *a priori* but was not prospectively registered. Although this does not alter the underlying trial data, lack of prospective registration reduces transparency regarding preplanned review decisions and should be considered when interpreting the review process.

### Future research directions

Future studies should prioritize: (1) pragmatic, head-to-head comparative effectiveness trials against other commonly used TRD augmentation strategies and, where feasible, stratified analyses by oral antidepressant backbone; (2) longer follow-up emphasizing sustained functioning, cognition, quality of life, and health-economic outcomes; (3) deeper evaluation of older adults and medically complex populations; and (4) better characterization of predictors of benefit (clinical phenotypes, biomarkers, and early response patterns) to support precision selection and reduce unnecessary exposure to adverse effects.

## Conclusions

Adjunctive intranasal esketamine plus an oral antidepressant provides rapid symptomatic improvement, increases the likelihood of response and remission, and reduces relapse risk in TRD, but it also increases acute adverse events—notably dissociation and blood-pressure increases—requiring structured monitoring. The overall benefit–harm profile supports its role as a targeted option within supervised care pathways, while highlighting the need for additional data in older adults, longer-term real-world outcomes, and backbone-specific comparative analyses.

## Data Availability

The original contributions presented in the study are included in the article/**Supplementary Material**. Further inquiries can be directed to the corresponding author.
