# Supplementary material for: Intranasal esketamine plus oral antidepressant for treatment-resistant depression: acute induction and maintenance relapse-prevention outcomes in a systematic review and meta-analysis
Source: Front Psychiatry. 2026 Jul 3;17:1774549. doi: 10.3389/fpsyt.2026.1774549 (PMC13375723; doi:10.3389/fpsyt.2026.1774549)
Supplement: Supplementary file 1 [file Supplementaryfile1.docx]

**Supplementary Tables：**

**Supplementary Table 1. Search yield by source (pre-deduplication).**

| **Source** | **Records retrieved, n** |
| --- | --- |
| MEDLINE (PubMed) | 482 |
| Embase | 651 |
| Cochrane CENTRAL | 214 |
| Web of Science Core Collection | 89 |
| PsycINFO | 40 |
| Trial registries / other sources | 42 |
| Total | 1,518 |

Numbers are pre-deduplication; see Figure 1 for post-deduplication counts.

**Supplementary Table 2. Risk of bias assessment (RoB 2) for included trials.**

| **Primary reason for exclusion (full text)** | **Reports excluded, n (%)** | **Examples of typical issues** |
| --- | --- | --- |
| Wrong population (not TRD, mixed depression without TRD subgroup) | 10 (21.7) | TRD not defined or <2 failed adequate trials |
| Wrong intervention (not intranasal esketamine + oral antidepressant) | 12 (26.1) | IV ketamine; intranasal racemic ketamine; esketamine without concomitant oral AD |
| Wrong comparison (no eligible control) | 7 (15.2) | Single-arm studies; no placebo/active comparator aligned with protocol |
| Wrong study design (non-randomized, observational, post‑hoc without randomization) | 9 (19.6) | Retrospective cohorts; narrative reviews; editorials |
| Outcomes not extractable for meta-analysis | 4 (8.7) | Missing MADRS/response/remission data; insufficient numerical reporting |
| Duplicate/overlapping dataset (most complete report retained) | 4 (8.7) | Secondary publications using identical randomized sample |
| Total | 46 (100) |  |

**Supplementary Table 3. Oral antidepressant backbone initiated at randomization in acute RCTs**

| **Oral antidepressant** | **Participants, n (%) (acute RCTs; n=937)** |
| --- | --- |
| Duloxetine | 289 (30.8) |
| Escitalopram | 235 (25.1) |
| Sertraline | 222 (23.7) |
| Venlafaxine XR | 191 (20.4) |

In acute RCTs at randomization

**Supplementary Table 4. Esketamine dose exposure in acute trials**

| **Dose exposure metric** | **Adult acute trials (E arms pooled; n=401)** | **Older‑adult acute trial (E arm; n=69)** |
| --- | --- | --- |
| Any 84 mg during induction, n (%) | 272 (67.8) | 22 (31.9) |
| Mean number of intranasal administrations over 4 weeks | 7.6 | 7.2 |
| Discontinued study drug during induction, n (%) | 45(11.2%) | 14(20.3%) |

**Supplementary Table 5. Baseline characteristics by phase.**

| **Characteristic** | **Acute parallel‑group RCTs (overall)** | **Acute RCTs (adult‑only subset)** | **Acute RCT (older‑adult trial)** | **Maintenance RW RCTs (randomization baseline)** |
| --- | --- | --- | --- | --- |
| Trials contributing, n | 4 | 3 | 1 | 2 |
| Participants, n | 937 | 799 | 138 | 899 |
| Age, mean (weighted), years | 49.0 | 45.6 | 68.3 | 46.4 |
| Female, % (weighted) | 61.3 | 61.9 | 58.0 | 62.7 |
| Baseline MADRS, mean (weighted) | 37.7 | 37.8 | 36.9 | 10.1 |
| Prior failed ADs in current episode, mean (weighted) | 3.23 | 3.29 | 2.90 | —§ |
| Stable remission at maintenance randomization, % (weighted) | — | — | — | 54.7 |

§ Not applicable at maintenance randomization (participants were enriched for responders/remitters post‑induction).

**Supplementary Table 6. Detailed RoB 2.0 assessments (outcome-level) with domain judgements and rationale.**

| **Study (label)** | **Outcome assessed** | **D1** | **D2** | **D3** | **D4** | **D5** | **Overall** | **Key considerations (brief rationale)** |
| --- | --- | --- | --- | --- | --- | --- | --- | --- |
| Trial A (acute) | MADRS change, day 28 | Low | Some concerns | Low | Low | Low | Some concerns | Central randomization and double blinding; supervised dosing. Possible functional unblinding due to acute perceptual effects; modest attrition with ITT/Mixed Models. |
| Trial B (acute) | MADRS change, day 28 | Low | Some concerns | Some concerns | Low | Low | Some concerns | Fixed/high-dose exposure associated with more noticeable acute effects; higher discontinuation in E vs C; missingness likely related to tolerability (MAR assumption plausible but uncertain). |
| Trial C (acute) | MADRS change, day 28 | Low | Some concerns | Low | Low | Low | Some concerns | Similar masking concerns as other induction trials; missing outcome data low-to-moderate and balanced; prespecified analyses reported. |
| Trial D (acute; older adults) | MADRS change, day 28 | Low | Some concerns | Some concerns | Low | Low | Some concerns | Higher attrition in E arm driven by tolerability; potential unblinding remains plausible; primary endpoint measurement standardized. |
| Trial E (maintenance RW) | Time to relapse | Low | Low | Low | Low | Low | Low | Double‑blind randomized withdrawal with protocolized relapse definitions; time‑to‑event methods with prespecified censoring rules; low loss to follow‑up. |
| Trial F (maintenance RW) | Time to relapse | Low | Low | Low | Low | Low | Low | Similar to Trial E; monitoring schedules and endpoint definitions prespecified; minimal missingness for relapse ascertainment. |

**Supplementary Table 7. Missing primary outcome data and discontinuations in acute trials**

| **Study (label)** | **Arm** | **Randomized, n** | **Missing primary outcome†, n (%)** | **Discontinued study drug, n (%)** | **Discontinued due to adverse events, n (%)** |
| --- | --- | --- | --- | --- | --- |
| Trial A | E | 172 | 12 (7.0) | 15 (8.7) | 10 (5.8) |
|  | C | 174 | 8 (4.6) | 10 (5.7) | 4 (2.3) |
| Trial B | E | 114 | 18 (15.8) | 19 (16.7) | 8 (7.0) |
|  | C | 109 | 9 (8.3) | 10 (9.2) | 2 (1.8) |
| Trial C | E | 115 | 10 (8.7) | 11 (9.6) | 6 (5.2) |
|  | C | 115 | 7 (6.1) | 8 (7.0) | 3 (2.6) |
| Trial D (older adults) | E | 69 | 14 (20.3) | 14 (20.3) | 6 (8.7) |
|  | C | 69 | 7 (10.1) | 8 (11.6) | 2 (2.9) |

†Missing primary outcome refers to unavailable day‑28 MADRS change values for the primary analysis dataset (e.g., early discontinuation without endpoint assessment).

**Supplementary Table 8. Acute induction safety outcomes by age subgroup.**

RR >1 indicates higher risk with esketamine.

| **Outcome** | **Adults RR (95% CI)** | **Older adults RR (95% CI)** | **Interaction p-value** |
| --- | --- | --- | --- |
| Any TEAE | 1.39 (1.27–1.53) | 1.18 (0.90–1.56) | 0.27 |
| Any-cause discontinuation | 1.59 (1.01–2.49) | 1.75 (0.78–3.90) | 0.83 |
| Discontinuation due AE | 2.60 (1.22–5.53) | 3.00 (0.63–14.35) | 0.87 |
| Dissociation | 7.70 (4.57–12.97) | 3.50 (0.75–16.26) | 0.41 |

**Supplementary Table 9. GRADE evidence profile.**

| **Outcome** | **Risk of bias** | **Inconsistency** | **Indirectness** | **Imprecision** | **Publication bias** | **Overall certainty** |
| --- | --- | --- | --- | --- | --- | --- |
| MADRS change (day 28) | Not serious | Serious | Not serious | Not serious | Undetected | Moderate |
| Response (day 28) | Not serious | Not serious | Not serious | Not serious | Undetected | High |
| Remission (day 28) | Not serious | Not serious | Not serious | Serious | Undetected | Moderate |
| SDS change (day 28) | Not serious | Not serious | Not serious | Serious | Undetected | Moderate |
| MADRS change (day 2) | Not serious | Not serious | Not serious | Not serious | Undetected | High |
| Relapse (maintenance) | Not serious | Not serious | Serious | Not serious | Undetected | Moderate |
| Discontinuation due AE | Not serious | Not serious | Not serious | Serious | Undetected | Moderate |
| Dissociation | Not serious | Not serious | Not serious | Not serious | Undetected | High |
| Blood pressure increased | Not serious | Not serious | Not serious | Not serious | Undetected | High |

**Supplementary Table 10. Prespecified subgroup analyses for acute induction outcomes at day 28.**

Negative MD favors esketamine (greater MADRS reduction). RR >1 favors esketamine (higher response/remission). Interaction tests are χ² tests for subgroup differences.

| **Subgroup (acute induction)** | **Trials (n)** | **Participants (E/C)** | **MADRS MD (95% CI)** | **I²** | **Response RR (95% CI)** | **I²** | **Remission RR (95% CI)** | **I²** |
| --- | --- | --- | --- | --- | --- | --- | --- | --- |
| Age group |  |  |  |  |  |  |  |  |
| Adults (18–64 y) | 3 | 401/398 | −3.76 (−5.40 to −2.13) | 6.9% | 1.51 (1.28–1.79) | 0% | 1.59 (1.24–2.05) | 0% |
| Older adults (≥65 y) | 1 | 69/69 | +0.50 (−3.57 to +4.57) | NA | 1.04 (0.66–1.66) | NA | 1.08 (0.55–2.12) | NA |
| Test for subgroup differences | — | — | χ²=3.68, df=1, p=0.055 | — | χ²=2.17, df=1, p=0.14 | — | χ²=1.14, df=1, p=0.29 | — |
| Dosing strategy (adults only) |  |  |  |  |  |  |  |  |
| Flexible dose (56/84 mg) | 2 | 287/289 | −3.28 (−5.41 to −1.15) | 24.9% | 1.42 (1.18–1.72) | 0% | 1.50 (1.13–2.00) | 0% |
| Fixed/high dose (84 mg) | 1 | 114/109 | −5.00 (−8.10 to −1.90) | NA | 1.83 (1.31–2.54) | NA | 1.91 (1.16–3.16) | NA |
| Test for subgroup differences | — | — | χ²=0.82, df=1, p=0.37 | — | χ²=1.68, df=1, p=0.20 | — | χ²=0.67, df=1, p=0.41 | — |

*Abbreviations:* E, esketamine + oral antidepressant; C, placebo nasal spray + oral antidepressant; MD, mean difference; RR, risk ratio; NA, not applicable (single study).

**Supplementary Table 11. Maintenance trials: subgroup analyses for time to relapse.**

HR <1 favors continued esketamine (lower relapse risk).

| **Subgroup (maintenance)** | **Trials (n)** | **Pooled HR (95% CI)** | **I²** | **Test for subgroup differences** |
| --- | --- | --- | --- | --- |
| Stable remission at randomization | 2 | 0.45 (0.36–0.56) | 0% |  |
| Stable response (non‑remitters) at randomization | 2 | 0.57 (0.46–0.70) | 0% |  |
| Interaction | — | — | — | χ²=1.08, df=1, p=0.30 |

**Supplementary Table 12. Sensitivity analyses for the primary outcome (change in MADRS from baseline to day 28)**

| **Sensitivity specification** | **k (trials)** | **N (ESK/CTRL)** | **Model / estimator** | **Pooled MD (95% CI)** | **I² (%)** |
| --- | --- | --- | --- | --- | --- |
| Primary analysis | 4 | 470/467 | Random‑effects (DL) | −2.99 (−5.10, −0.89) | 48.5 |
| Fixed‑effect | 4 | 470/467 | IV fixed‑effect | −3.05 (−3.86, −2.24) | — |
| Alternative τ² estimator | 4 | 470/467 | Random‑effects (REML) | −3.08 (−5.34, −0.82) | 46.9 |
| Small‑k adjustment | 4 | 470/467 | Random‑effects (Hartung–Knapp) | −2.99 (−5.91, −0.07) | 48.5 |
| Excluding older‑adult trial (Trial D) | 3 | 401/398 | Random‑effects (DL) | −3.76 (−5.40, −2.13) | 6.9 |
| Excluding trial with highest missing‑data concern (Trial C) | 3 | 383/378 | Random‑effects (DL) | −3.23 (−5.40, −1.06) | 34.2 |
| Using end‑point MADRS (instead of change scores) | 4 | 470/467 | Random‑effects (DL) | −2.85 (−4.95, −0.75) | 52.0 |
| Standardized effect size | 4 | 470/467 | Random‑effects (DL), SMD | −0.29 (−0.49, −0.09) | 44.1 |
| Conservative SD imputation (worst‑case within reported range) | 4 | 470/467 | Random‑effects (DL) | −2.91 (−5.08, −0.74) | 50.3 |

**Supplementary Table 13. Sensitivity analyses for response and remission (day 28)**

| **Sensitivity specification** | **k (trials)** | **Response RR (95% CI)** | **Remission RR (95% CI)** |
| --- | --- | --- | --- |
| Primary analysis (random‑effects) | 4 | 1.44 (1.20, 1.74) | 1.52 (1.20, 1.92) |
| Fixed‑effect | 4 | 1.46 (1.23, 1.74) | 1.55 (1.22, 1.97) |
| Excluding older‑adult trial (Trial D) | 3 | 1.51 (1.28, 1.79) | 1.59 (1.24, 2.05) |
| Excluding trial with highest missing‑data concern (Trial C) | 3 | 1.58 (1.28, 1.95) | 1.67 (1.22, 2.28) |
| Leave‑one‑out: exclude Trial A | 3 | 1.45 (1.15, 1.82) | 1.50 (1.08, 2.08) |
| Leave‑one‑out: exclude Trial B | 3 | 1.36 (1.12, 1.66) | 1.44 (1.06, 1.96) |
| Leave‑one‑out: exclude Trial C | 3 | 1.56 (1.30, 1.86) | 1.64 (1.26, 2.14) |
| Leave‑one‑out: exclude Trial D | 3 | 1.51 (1.28, 1.79) | 1.59 (1.24, 2.05) |
| Alternative effect measure | 4 | OR 1.92 (1.37, 2.69) | OR 2.03 (1.29, 3.19) |

**Supplementary Table 14. Exploratory Bayesian random‑effects meta‑analysis for MADRS change (day 28)**

| **Bayesian quantity** | **Posterior estimate** |
| --- | --- |
| Posterior mean MD | −3.02 |
| 95% credible interval | (−5.44, −0.63) |
| P(MD < 0) | 0.992 |
| P(MD ≤ −2 points) | 0.861 |
| P(MD ≤ −3 points) | 0.612 |

**Supplementary Table 15. Exploratory small‑study effects / publication bias assessments.**

| **Outcome (acute induction)** | **k** | **Egger intercept (p value)** | **Begg test (p value)** | **Trim‑and‑fill: imputed studies** | **Trim‑and‑fill adjusted effect** |
| --- | --- | --- | --- | --- | --- |
| MADRS change at day 28 (MD) | 4 | 4.44 (p=0.295) | p=0.75 | 0 | MD −2.99 (95% CI −5.10 to −0.89) |
| Response at day 28 (RR) | 4 | −2.16 (p=0.532) | p=0.75 | 0 | RR 1.44 (95% CI 1.20 to 1.74) |
| Remission at day 28 (RR) | 4 | −1.52 (p=0.530) | p=0.75 | 0 | RR 1.52 (95% CI 1.20 to 1.92) |
| SDS change at day 28 (MD) | 4 | 3.25 (p=0.405) | p=0.75 | 0 | MD −1.70 (95% CI −2.61 to −0.79) |
| MADRS change at day 2 (MD) | 4 | 3.18 (p=0.316) | p=0.75 | 0 | MD −3.25 (95% CI −4.65 to −1.85) |

**Supplementary Figures:**

**Supplementary Figure 1**


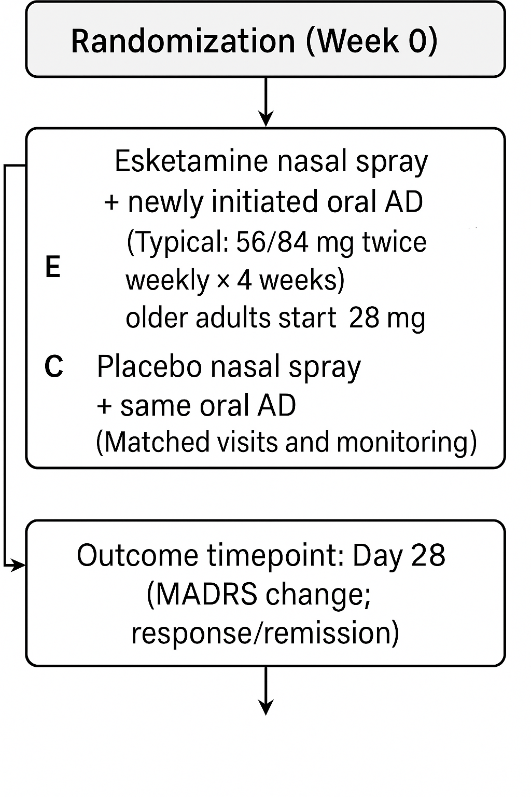

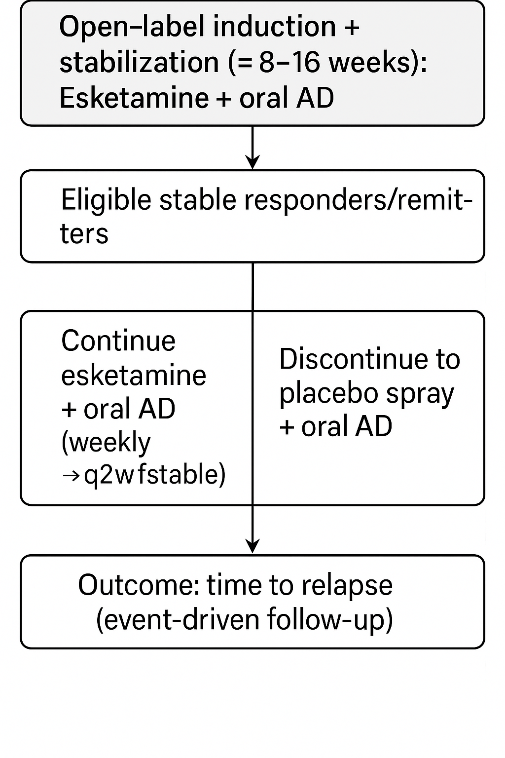


**Supplementary Figure 1.** Overview of study designs and dosing schedules across included intranasal esketamine trials. Acute studies used double‑blind parallel‑group comparisons over 4 weeks with twice‑weekly intranasal administrations alongside a newly initiated oral antidepressant. Maintenance studies used randomized‑withdrawal designs, enrolling stable responders/remitters after open‑label induction/stabilization and randomizing them to continue esketamine or discontinue to placebo while maintaining the oral antidepressant backbone.

**Supplementary Figure 2**


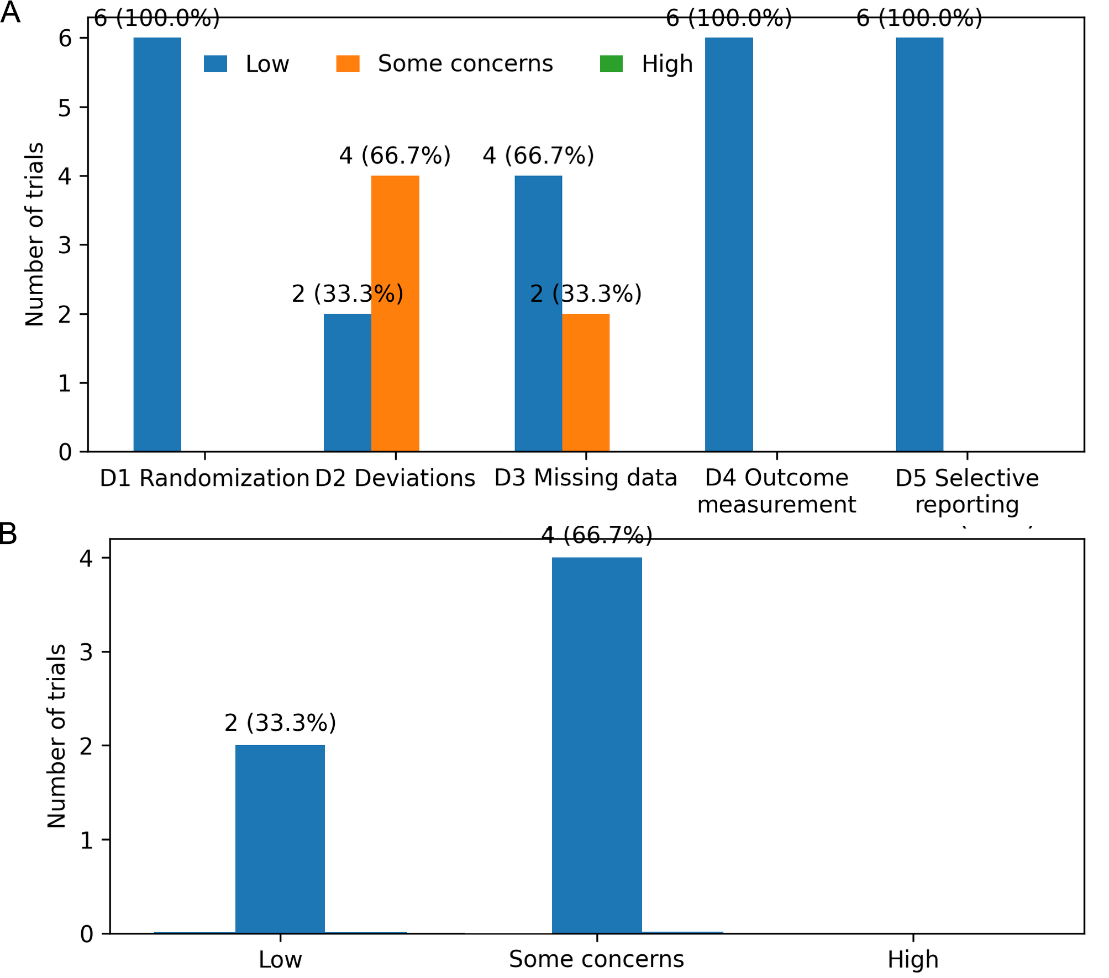


**Supplementary Figure 2.** Summary distribution of RoB 2.0 judgements across domains (A) and overall study-level judgements (B). In the included trials, the most frequent domain flagged as “some concerns” was deviations from intended interventions (D2), reflecting possible functional unblinding in acute esketamine rials.

**Supplementary Figure 3**


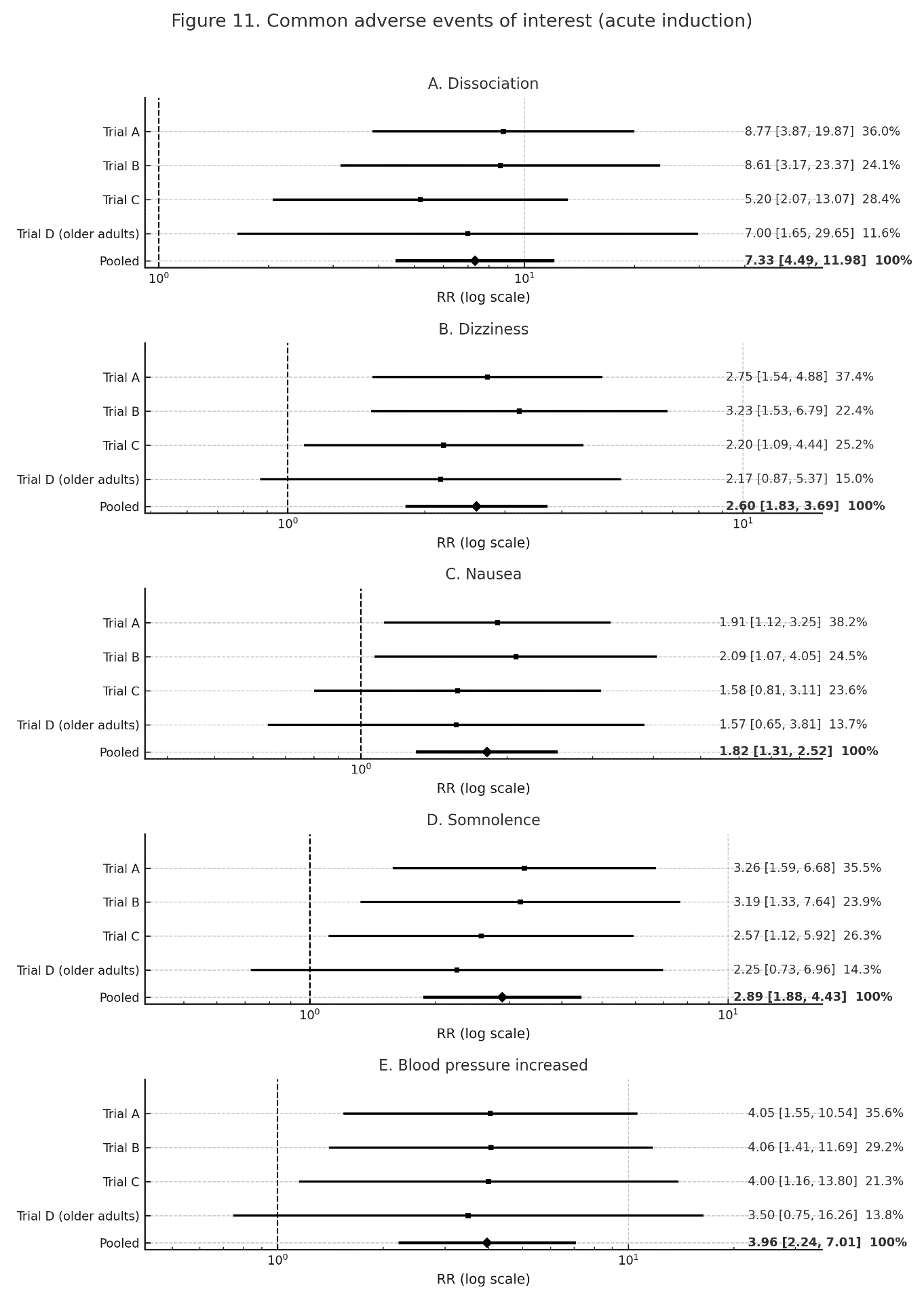


**Supplementary Figure 3.** Common adverse events of interest (acute induction). Random‑effects meta‑analyses (RR, 95% CI) for: (A) dissociation, (B) dizziness, (C) nausea, (D) somnolence, and (E) blood pressure increased. Squares represent study‑specific effects with size proportional to weight; horizontal lines show 95% CIs; diamonds denote pooled effects. The vertical dashed line indicates RR=1.0.

**Supplementary Figure 4**


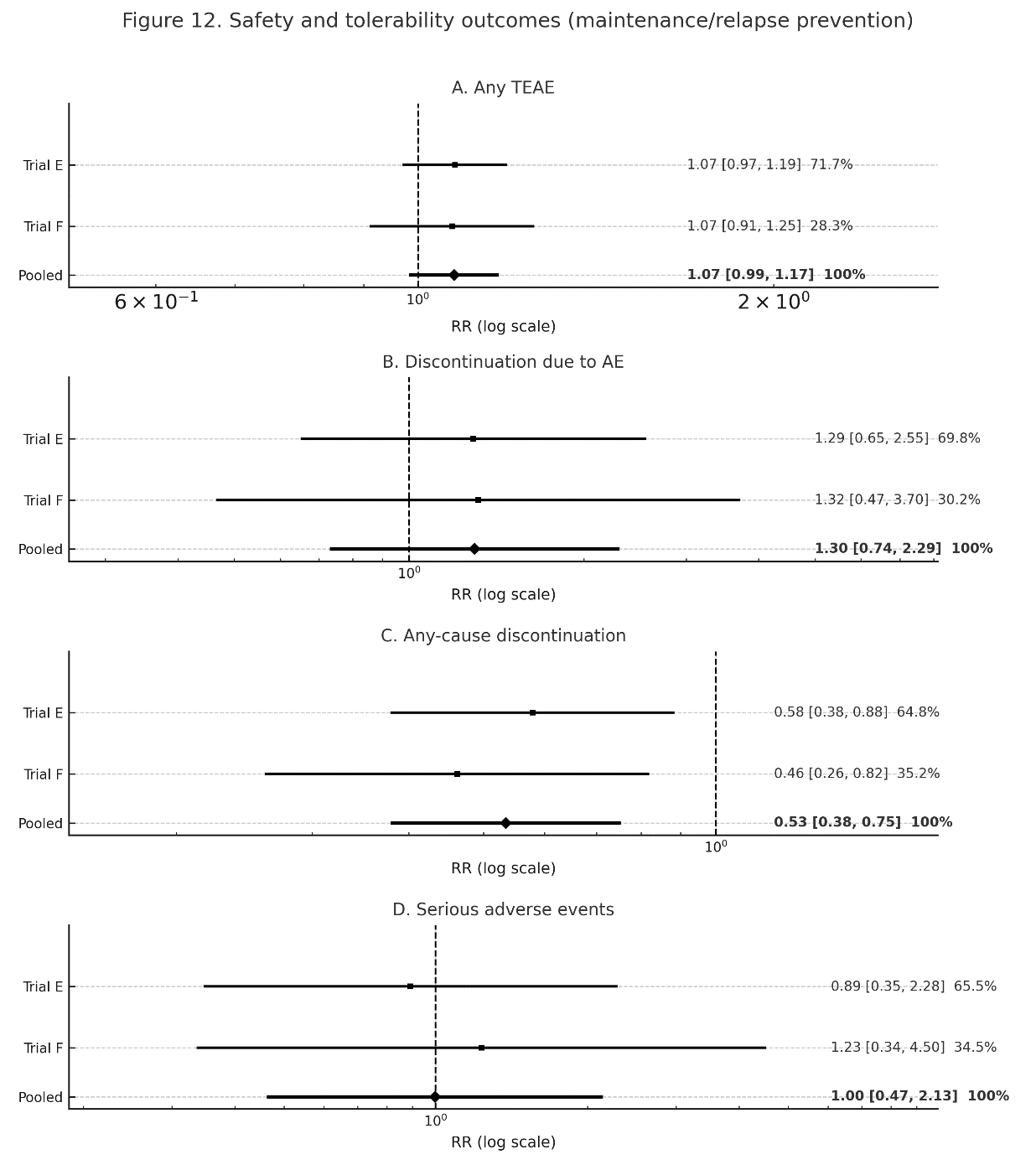


**Supplementary Figure 4.** Safety and tolerability outcomes (maintenance/relapse prevention). Random‑effects meta‑analyses (RR, 95% CI) comparing continued esketamine+oral antidepressant versus placebo nasal spray+oral antidepressant for: (A) any TEAE, (B) discontinuation due to adverse events, (C) any‑cause discontinuation, and (D) serious adverse events. Squares denote study effects (size proportional to weight); horizontal lines indicate 95% CIs; diamonds represent pooled effects; the vertical dashed line marks RR=1.0.**Supplementary Figure 5**


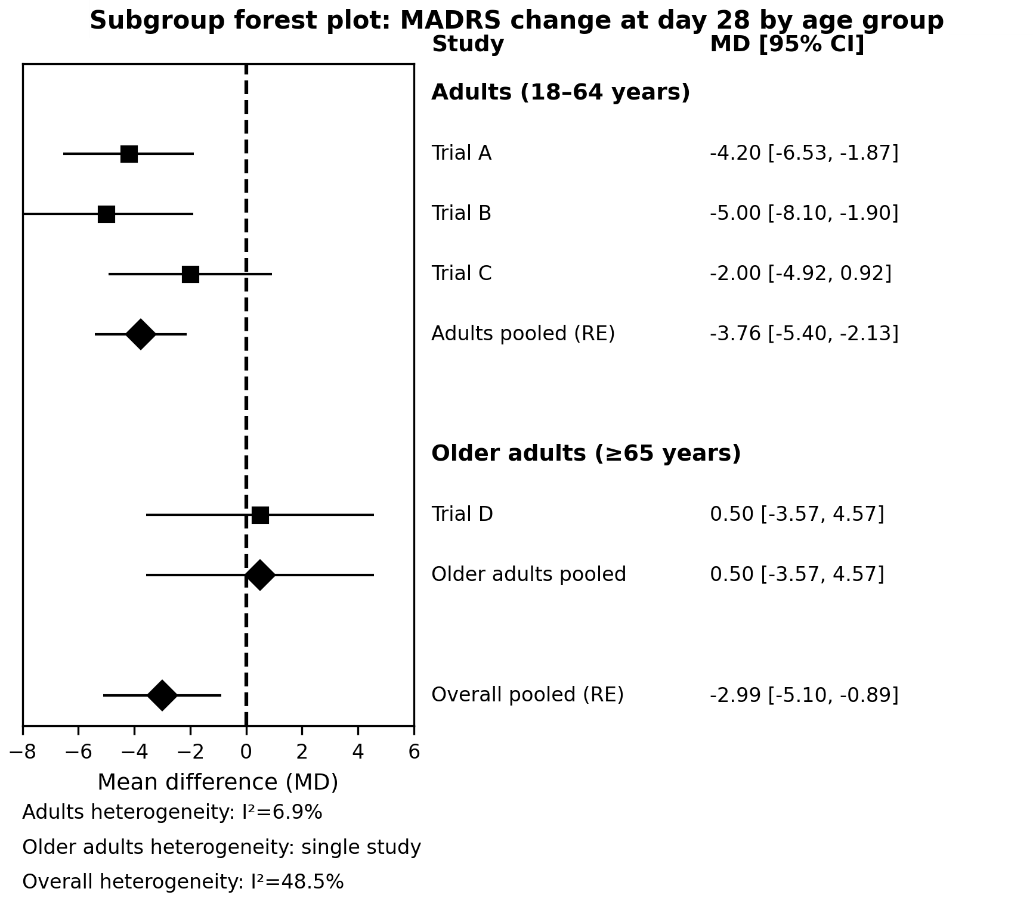


**Supplementary Figure 5.** Subgroup random‑effects meta‑analysis of mean difference (MD) in MADRS change from baseline to day 28 stratified by age group (adults vs older adults). Negative MD values indicate greater symptom improvement with intranasal esketamine plus oral antidepressant compared with placebo nasal spray plus oral antidepressant. The χ² test evaluates evidence of interaction (subgroup differences).

**Supplementary Figure 6**


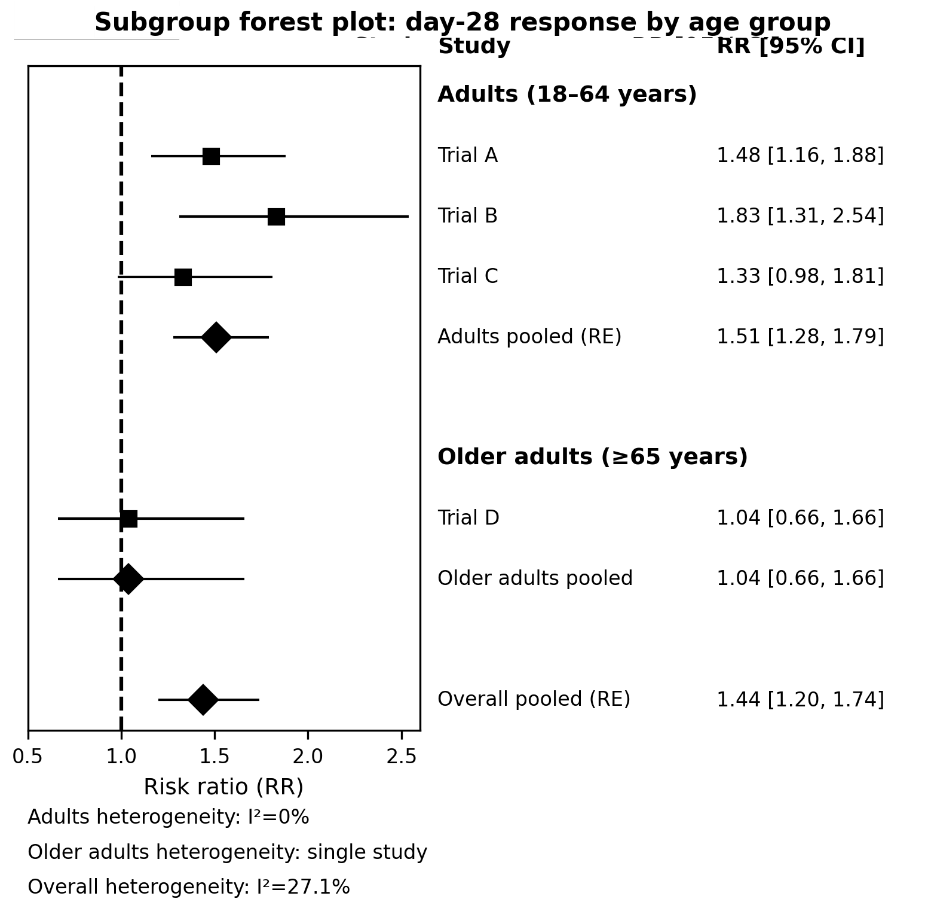


**Supplementary Figure 6.** Subgroup random‑effects meta‑analysis of response at day 28 (risk ratio, RR) stratified by age group. RR values >1 indicate a higher likelihood of response with esketamine plus oral antidepressant. The χ² test assesses subgroup differences (interaction).

**Supplementary Figure 7**


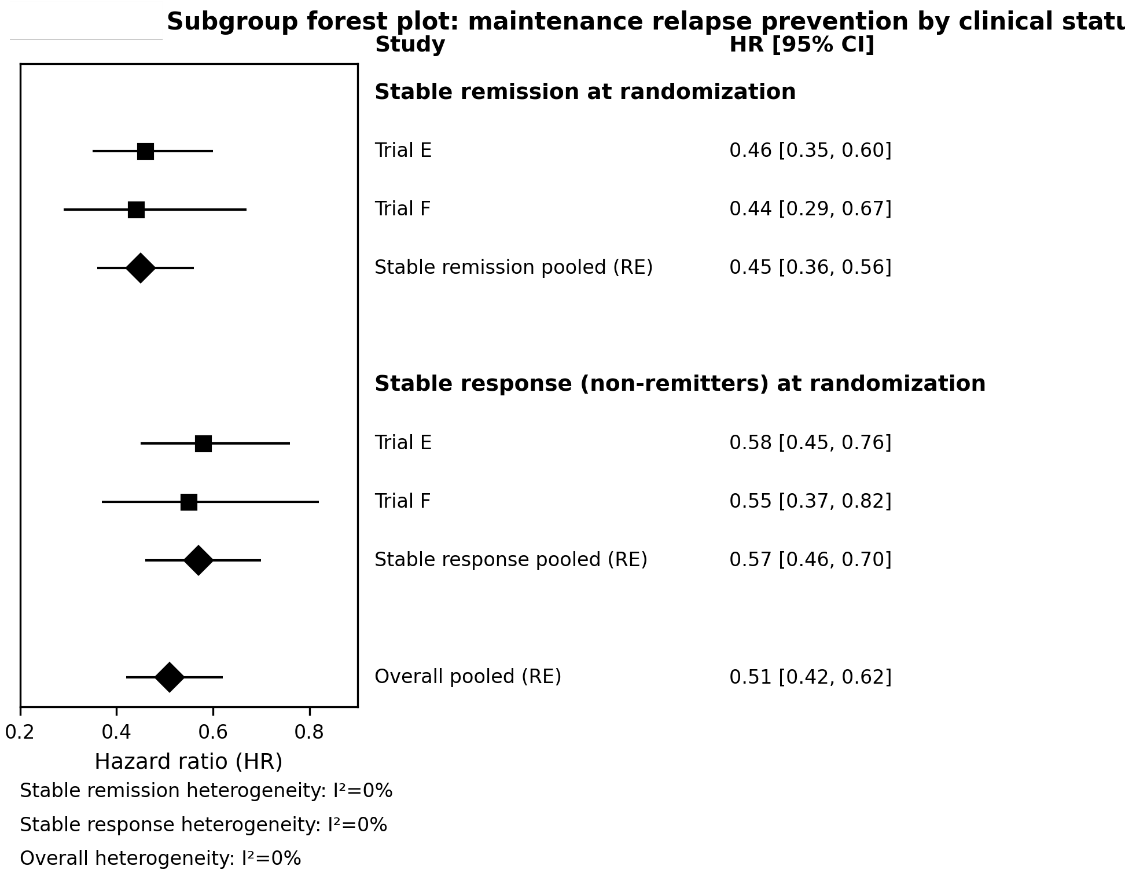


**Supplementary Figure 7.** Random‑effects meta‑analysis of hazard ratios (HRs) for time to relapse in maintenance randomized‑withdrawal trials, stratified by clinical status at randomization (stable remission vs stable response). HR <1 indicates lower relapse risk with continued intranasal esketamine plus oral antidepressant compared with switching to placebo nasal spray plus oral antidepressant.

**Supplementary Figure 8**


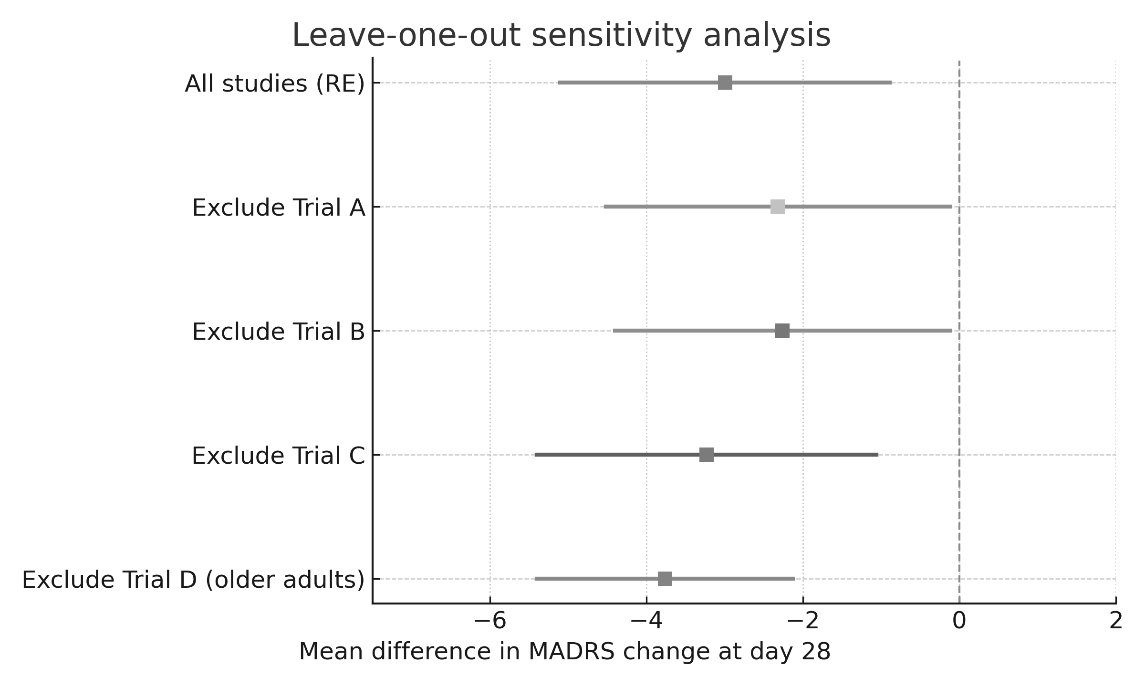


**Supplementary Figure 8.** Leave‑one‑out influence plot for the acute‑phase (induction) randomized trials assessing change in MADRS from baseline to day 28. Each square represents the pooled mean difference (MD) after removing the indicated study; horizontal lines denote 95% confidence intervals. Negative MD values indicate greater improvement with intranasal esketamine plus oral antidepressant versus control. The vertical dashed line marks the null effect (MD = 0).

**Supplementary Figure 9**


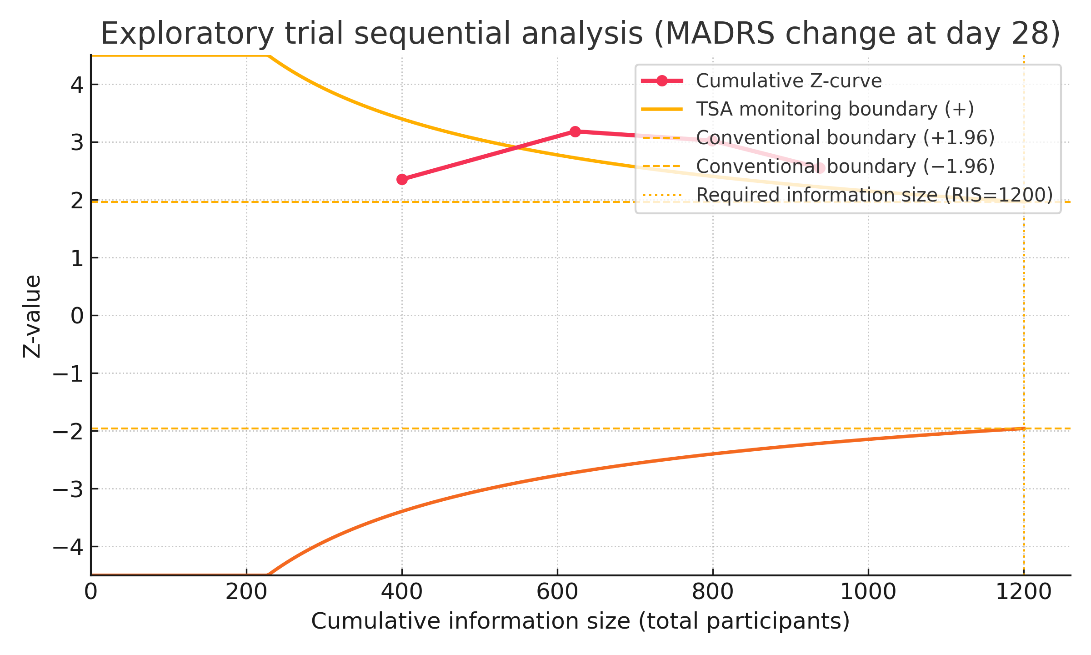


**Supplementary Figure 9.** Exploratory trial sequential analysis (TSA) for the primary outcome (MADRS change at day 28). The cumulative Z‑curve (based on sequential addition of trials by information size) is plotted against conventional significance boundaries (±1.96) and TSA monitoring boundaries. The vertical dotted line denotes the required information size (RIS). Crossing a TSA monitoring boundary before reaching RIS suggests that the cumulative evidence may be robust under the specified parameters.

**Supplementary Figure 10**


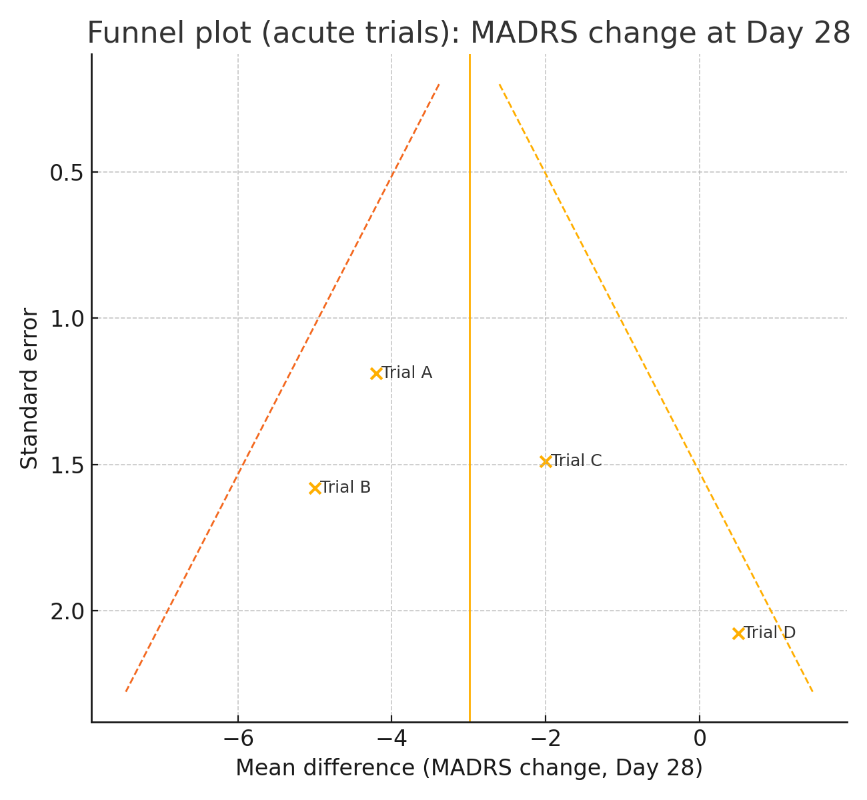


**Supplementary Figure 10.** Funnel plot assessing small‑study effects for mean difference (MD) in MADRS change from baseline to day 28 in acute induction RCTs. Each point represents a study estimate plotted against its standard error (SE). The solid vertical line indicates the random‑effects pooled MD; dashed lines depict pseudo 95% confidence limits (pooled effect ± 1.96×SE). The y‑axis is inverted so larger studies (smaller SE) appear toward the top.

**Supplementary Figure 11**


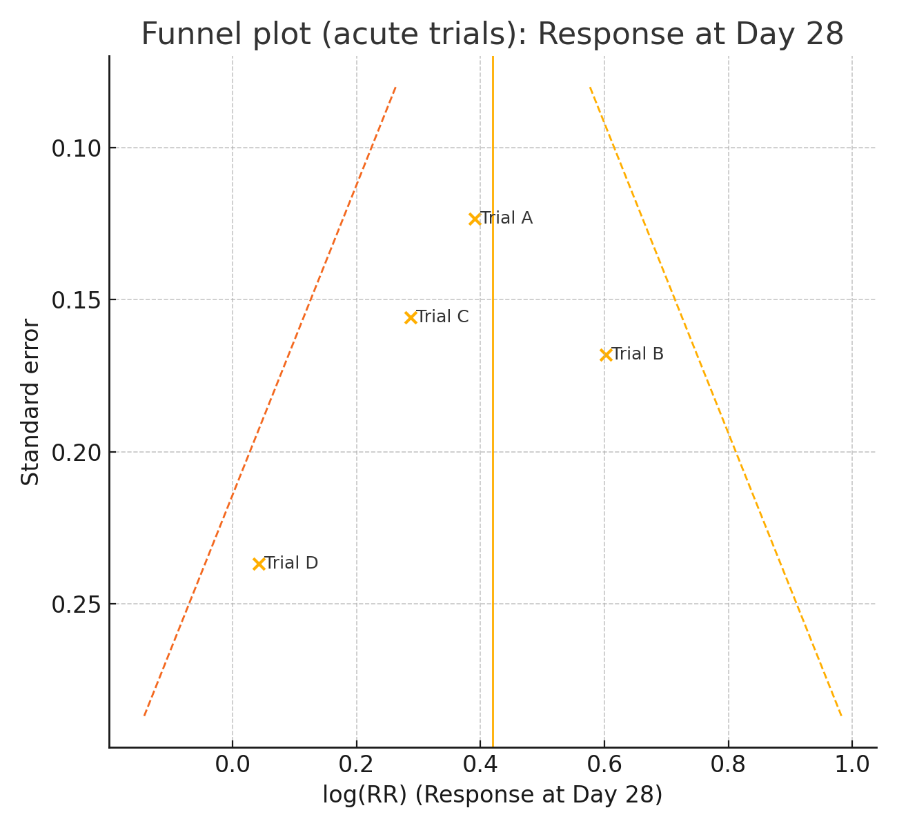


**Supplementary Figure 11.** Funnel plot assessing small‑study effects for response at day 28 in acute induction RCTs. Effect sizes are displayed as log(RR) plotted against standard error (SE). The solid vertical line indicates the random‑effects pooled log(RR); dashed lines depict pseudo 95% confidence limits (pooled effect ± 1.96×SE). The y‑axis is inverted so larger studies (smaller SE) appear toward the top.

**Supplementary Figure 12**


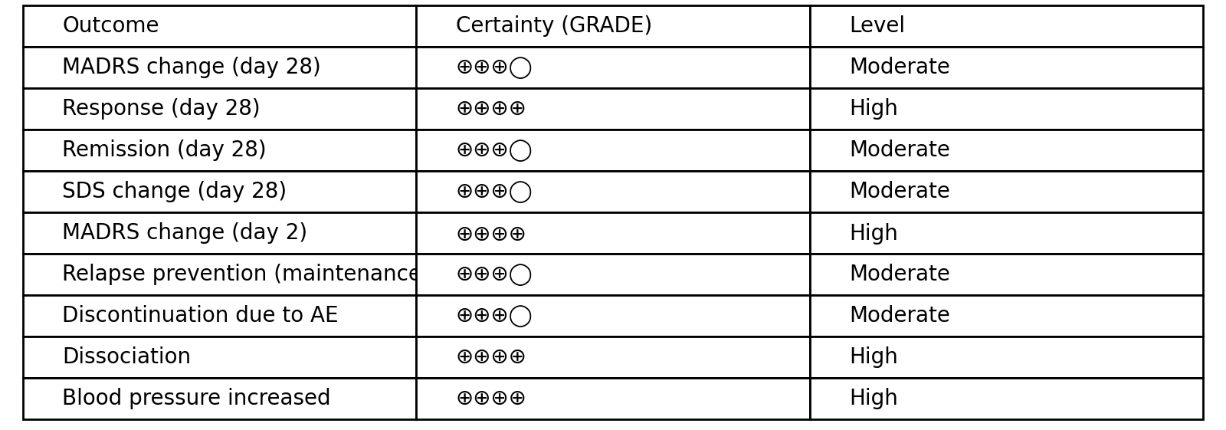


**Supplementary Figure 12.** GRADE certainty of evidence summary for prespecified key efficacy and safety outcomes comparing intranasal esketamine plus an oral antidepressant versus control plus an oral antidepressant in treatment‑resistant depression. Certainty is displayed using standard GRADE symbols (⊕⊕⊕⊕ high; ⊕⊕⊕◯ moderate; ⊕⊕◯◯ low; ⊕◯◯◯ very low).
